# Fermented Soybean Pulp Alleviates Disease Progression of 5×FAD Model Mice

**DOI:** 10.1007/s12035-025-05191-y

**Published:** 2025-07-15

**Authors:** Chun-Yen Yang, Yu-Hsuan Liu, Ta-Chun Lin, Kuo-Hsuan Chang, Hsiu Mei Hsieh-Li

**Affiliations:** 1 Department of Life Sciences, National Taiwan Normal University, Taipei, Taiwan; 2 Department of Neurology, Chang Gung Memorial Hospital, Chang Gung University College of Medicine, Taoyuan, Taiwan

**Keywords:** Alzheimer’s disease, 5 × FAD, Cognition, Fermented soybean pulp, AKT/GSK3β/NRF2, BDNF

## Abstract

Alzheimer’s disease (AD) is the most common neurodegenerative disorder of the central nervous system, characterized by memory loss and cognitive decline. The two main hypotheses regarding AD involve the accumulation of amyloid-β (Aβ) forming plaques and the intracellular hyperphosphorylation of tau protein, leading to the formation of neurofibrillary tangles (NFT). These processes are accompanied by neuroinflammation and oxidative stress, and eventual neuronal death. While soy foods are widely recognized for their nutritional benefits, soybean pulp (okara), the residue left over from making tofu or soybean milk, is mostly discarded as kitchen waste, despite being rich in nutrients such as dietary fiber, protein, and isoflavones. This underutilized byproduct may serve as a valuable resource for functional food development and sustainable resource use. In this study, fermented soybean pulp (FS) demonstrated neuroprotective effects. In vitro, FS at concentrations of 0.001 µg/mL and 0.01 µg/mL significantly improved cell viability in Aβ-induced HT-22 cells and reduced lipid peroxidation. Further, in vivo oral administration of FS attenuated the cognitive deficits of 5 × FAD mice, enhancing both short and long-term memory and reducing anxiety-like behaviors. Immunohistochemical analysis revealed that the FS-treated 5 × FAD mice group significantly reduced hippocampal amyloid plaque accumulation and gliosis. FS also upregulated the expression levels of brain-derived neurotrophic factor (BDNF), PSD95, and synaptophysin, while preventing hippocampal neuronal loss. Mechanistically, FS may activate the Nrf2 antioxidant pathway and NF-κB-mediated inflammation through the modulation of the Akt/GSK3β signaling axis in the hippocampus. These molecular actions likely contribute to increased antioxidant enzymes and suppressed neuroinflammatory responses. Overall, this study suggests that FS has therapeutic potential for alleviating cognitive and behavioral impairments in AD. Moreover, the repurposing of soybean pulp, which would otherwise be discarded, enhances its utilization value and supports sustainable green recycling.

**Supplementary Information:**

The online version contains supplementary material available at 10.1007/s12035-025-05191-y.

## Introduction

According to statistics from the World Health Organization, over 50 million people worldwide are affected by dementia. Alzheimer’s disease (AD) is the most common form, accounting for approximately 60–80% of all cases [1]. Early symptoms of AD include anxiety and apathy, progressing to memory loss and the inability to manage daily living activities [2]. Although the exact etiology of AD remains complex and multifactorial, two widely accepted pathological hypotheses involve the accumulation of amyloid-β (Aβ) and the hyperphosphorylation of tau protein [3, 4]. Additionally, increasing evidence has demonstrated that impaired antioxidant defense and neuroinflammation play a critical role in the pathogenesis and progression of AD [5, 6]. Both Aβ plaque and neurofibrillary tangles (NFTs) are known to trigger chronic neuroinflammatory responses and impair antioxidant defense systems, exacerbating neurotoxicity. These pathological processes ultimately result in acetylcholine depletion, synaptic dysfunction, and neuronal death [7].

Nuclear factor erythroid 2-related factor 2 (NRF2) is a key transcription factor that regulates inflammatory and oxidative stress [8]. NRF2 activates a variety of downstream antioxidant enzymes, including heme oxygenase-1 (HO-1), superoxide dismutase (SOD), and glutathione peroxidase (GSH-Px), which are essential for mitigating oxidative damage [9]. NRF2 also plays a crucial role in maintaining neuronal survival and promoting neuroplasticity [10, 11]. It has been observed in AD patients that a significant reduction in nuclear NRF2 expression in the CA1 region of the hippocampus [12] leads to decreased expression of genes of antioxidant enzymes and renders neurons more vulnerable to oxidative stress-induced damage [13]. Moreover, NRF2 deficiency has been shown to exacerbate cognitive impairments in AD mouse models [14, 15]. A key upstream regulator of NRF2 is the AKT/GSK3β signaling pathway [16]. Activated GSK3β inhibits NRF2 by preventing its nuclear translocation, promoting its ubiquitination, and targeting it for proteasomal degradation [17, 18]. Conversely, activation of the AKT signaling pathway leads to phosphorylation and inactivation of GSK3β, thereby preventing its inhibitory effect on NRF2 [19]. Various compounds have been reported to influence the AKT/GSK3β signaling, leading to NRF2 activation and mitigation of AD-related symptoms [20–22]. It suggests that the AKT/GSK3β/NRF2 pathway may offer a promising therapeutic strategy for AD treatment.

Fermentation has been used to enhance the bioavailability of minerals, vitamins, and isoflavones within soy products [23, 24]. It also reduces the levels of anti-nutritional factors, such as protease inhibitors, phytic acid, and phenolic compounds, thereby improving nutritional value and facilitating nutrient absorption [25, 26]. Recent studies have shown that various fermented soy products exhibit beneficial effects in animal models of neurodegenerative diseases [27]. Fermentation with *Bacillus licheniformis* enhances cognitive function and blood sugar regulation in AD and type 2 diabetes rats [28]. Similarly, fermented soybean products such as Cheonggukjang, produced using *Bacillus subtilis* MC31 and *Lactobacillus sakei* 383, were found to increase nerve growth factor (NGF) levels and SOD activity, leading to improved cognitive performance in mice [29]. In addition to preclinical findings, clinical studies have further highlighted the neuroprotective potential of fermented soy. A notable example is DW2009, a product of soybean fermented by *Lactobacillus plantarum* C29, which enhanced cognitive function in individuals with mild cognitive impairment, associated with a significant correlation between cognitive improvement and serum BDNF level [30].

These findings suggest the potential protective effects of fermented soybeans against AD. FS used in this study is rich in genistein, an isoflavone known for its neuroprotective properties, including the reduction of Aβ aggregation, suppression of inflammation, enhancement of antioxidant activity, and modulation of stress behavior [31–33]. Additionally, fermented products are rich in antioxidants and various bioactive compounds, which may influence brain function through the gut-brain axis [34, 35]. Based on these observations, we hypothesize that FS has the potential to prevent neuronal damage and slow the progression of neurodegenerative disorders. This study investigated the potential of FS in preventing or mitigating AD using HT-22 hippocampal neuronal cell lines and 5 × FAD transgenic mice. In addition, the underlying molecular mechanisms of FS-mediated neuroprotection were systematically investigated.

## Materials and Methods

### Preparation of FS

Soybean pulp was mixed with a *Levilactobacillus brevis* BCRC12310 solution (10^6^ CFU/mL) at a ratio of 15:1 (w/v) and homogenized for 3 min. The homogenized mixture (150 g) was placed into a sterilized 5-L glass flask and incubated for 24 h at 30 °C. After fermentation, the soybean pulp was dried using a drum dryer. To prepare the solution, 0.1 g of FS was dissolved in 10 mL of PBS, centrifuged at 160 × *g* for 10 min, and the supernatant was collected and diluted to the required concentrations for experiments.

### Composition Analysis of FS

Determination of isoflavone contents was performed as previously reported [36]. Briefly, 100 mg of FS was extracted with 1.0 mL of acetone/water/acetic acid (70:29.5:0.5, v/v/v) at 30 °C for 3 h with continuous shaking. The extract was then centrifuged at 12,000 × *g* at 4 °C for 20 min. The resulting supernatant was subjected to HPLC analysis using a C18 column. Gradient elution was conducted using water and acetonitrile at a flow rate of 2 mL/min, with detection at 262 nm. Determination of γ-aminobutyric acid (GABA) content was conducted based on the method of Rossetti and Lombard [37], with minor modifications (manuscript in preparation). Briefly, 100 mg of FS was extracted with 1.0 mL of 70% ethanol by shaking for 30 min. The mixture was then centrifuged at 1530 × *g* for 20 min. The supernatant was filtered through a 0.22-µm nylon filter and analyzed by HPLC using an Inertsil ODS-2 C18 column. Isocratic elution was performed at a flow rate of 0.6 mL/min for 30 min, and detection was carried out at 254 nm using a UV/VIS detector. Anthocyanin content was analyzed according to Huang et al. [38]. Cellulose content was determined according to the AOAC method [39].

### Cell Cultures

HT-22 is a mouse hippocampal neuronal cell line derived from differentiated HT-4 mouse hippocampal progenitor cells. HT-22 cells are increasingly recognized as a valuable model for studying AD, particularly in relation to neurotoxicity and cholinergic signaling [40, 41]. The primary components of Aβ plaques in neuronal cells are Aβ_1-40_ and Aβ_1-42_, both of which contain the Aβ_25-35_ fragment [42, 43]. Aβ_25-35_ is known to self-aggregate [42] and exert cytotoxic effects [44, 45]. Therefore, this study uses aggregated oligomeric Aβ_25-35_ (AnaSpec, USA) to induce neurotoxicity in HT-22 cells. HT-22 cells (3 × 10^3^) were seeded into each well of a 96-well plate and cultured for 22 h. Afterward, FS was then added, and the cells were cultured for an additional 2 h. Cell viability was assessed at 24 and 48 h post-Aβ_25-35_ (20 µM) addition using the MTT assay (Sigma-Aldrich, USA). MTT solution (0.5 mg/mL) was added and incubated at 37 °C for 2 h. The medium was then removed, and 100 µL of DMSO (Sigma-Aldrich, USA) was added to dissolve the formazan crystals. Absorbance was measured at 570 nm using a microplate reader (Thermo Fisher, USA) to determine cell viability.

### NAD +/NADH Assay

The NAD/NADH levels in HT-22 cells were measured using a commercial NAD/NADH assay kit (Abcam, UK) according to the manufacturer’s instructions. NAD (nicotinamide adenine dinucleotide) is a crucial coenzyme that acts as an electron carrier in the tricarboxylic acid cycle to the electron transport chain for ATP production. Therefore, the NAD⁺/NADH ratio reflects mitochondrial activity [46].

### Lipid Peroxidation Assay

Lipid peroxides are oxidative byproducts formed when reactive oxygen species (ROS) generate free radicals that oxidize lipids [47]. The oxidation of polyunsaturated fatty acids (PUFAs) generates malondialdehyde (MDA), a well-characterized and stable product of lipid peroxidation. MDA is widely recognized as a biomarker for oxidative damage, with its accumulation reflecting the extent of lipid oxidation and cellular oxidative stress in AD [48, 49]. Lipid peroxidation levels in HT-22 cells were analyzed using an MDA assay kit (Abcam, UK). For each group, 2 × 10⁶ cells were lysed in 303 µL of Lysis Solution (300 µL of Lysis Buffer with 3 µL of BHT). The lysates were homogenized and centrifuged at 13,000 × *g* at 4 °C for 10 min to collect the supernatant. The supernatant was then mixed with 600 µL of TBA solution and heated at 95 °C for 1 h. The resulting solution was transferred to a 96-well plate, and absorbance was measured at 532 nm to quantify lipid peroxidation.

### Protein Carbonyl Content Assay

Protein carbonylation is an irreversible oxidative modification that primarily affects the side chains of proline, lysine, arginine, and valine residues, resulting in the formation of carbonyl groups (− CO) within proteins [50]. As a hallmark of protein oxidation, carbonyl content is widely used to evaluate the extent of oxidative damage [51]. Protein carboxylation in HT-22 cells was measured using a Protein Carbonyl Content Assay Kit (Abcam, UK). According to the manufacturer’s instructions, colorimetric analysis was performed using a microplate reader that measures absorbance at 375 nm.

### Neurite Outgrowth Staining

Neurite outgrowth in HT-22 cells was analyzed using the Neurite Outgrowth Staining kit (Thermo Fisher, USA). A total of 1500 cells were seeded into each well of a 96-well plate and cultured for 36 h. After the medium was removed, the cells were washed three times with PBS and fixed with PFA for 30 min. Following three additional PBS washes, the working stain solution was applied to the cells and incubated at room temperature for 20 min. The stain solution was then removed, and the nuclei were stained with DAPI (1:10,000 in PBS) for 5 min. After removing DAPI, background suppression dye was added. Fluorescent images were captured using a fluorescence microscope, and neurite outgrowth was quantified using MetaXpress software (Molecular Devices, USA).

### Experimental Animals

This experiment used the 5 × FAD mouse model, B6.Cg-Tg (APPSwFlLon,PSEN1*M146L*L286V) 6799Vas) purchased from Jackson Laboratory (#008703; ME, USA). Wild-type (C57BL/6) mice were obtained from the National Laboratory Animal Center (Taipei, Taiwan). The 5 × FAD mouse model carries three mutations in the APP genes, K670N/M671L (Swedish), I716V (Florida), and V717I (London), and two in the PS1 genes, M146L and L286V, which collectively facilitate the accumulation of Aβ and result in early onset of AD pathology [52]. Amyloid plaques appear in the mouse hippocampus by 2 months of age, following the short-term and spatial memory deficits emerging between 3 and 6 months [53, 54]. All experiments were conducted on 5-month-old 5 × FAD and wild-type mice. Animals were grouped based on body weight and balanced for sex (*n* = 12/group). Four groups were established: wild-type mice with saline (WT + S), wild-type mice with FS (WT + FS), 5 × FAD with saline (TG + S), and 5 × FAD with FS (TG + FS). After 1-week acclimatization, mice were administered orally 4.2 mg/0.1 mL FS or 0.1 mL saline (0.9% NaCl) for 57 days. The dosage of FS was determined based on previous studies using fermented extracts in mice, in which an oral dose of 200 mg/kg was commonly applied [55–57]. This was converted to a daily dose of 4.2 mg for the mouse, formulated in 0.1 mL for oral administration. Behavioral tasks, including open field test (OFT), Barnes maze 1 (BM1), Y maze, elevated plus test (EPM), and BM2, were performed on days 45–55. Mice were sacrificed on day 57. Body weight was measured at five time points during the experiment. All of the animal experiments were conducted according to the Institutional Animal Care and Use Committee (IACUC) of the National Taiwan Normal University, Taipei, Taiwan (Permit number: NTNU112017), and all effort was made to minimize animal suffering. All behavioral data were collected and analyzed by EthoVision XT (Noldus Information Technology, Netherlands).

### Open Field Test (OFT)

The OFT was conducted to assess motor activity and anxiety-related behavior as described by Lee et al. [58] and Chiang et al. [59]. Mice were habituated to the testing room in their home cages for 30 min before testing. Each mouse was then individually placed in the center of a white acrylic box (30 × 30 × 30 cm) and allowed to explore freely for 10 min under 6 lx illumination. The box was divided into central (15 × 15 cm) and peripheral areas. During the first 5 min, the total distance traveled by each mouse was measured as an indicator of locomotor activity. In the subsequent 5 min, the time each mouse spent in the central area was recorded to assess its anxiety level.

### Elevated Plus Maze (EPM)

The EPM test was conducted to assess the anxiety behavior based on mice’s aversion to open elevated spaces. The maze was elevated 50 cm above the ground and consisted of two open arms (L 30 cm, W 5 cm), two enclosed arms (L, 30 cm; W, 5 cm; H, 15 cm), and a central platform (L, 5 cm; W, 5 cm). The task was performed as per previous report [59], with each mouse individually placed at the center and allowed to explore freely for 5 min. The time spent in the open arms was recorded as an anxiolytic level [60, 61].

### Y Maze

The Y maze test was performed to evaluate short-term memory based on mice’s natural preference for exploring novel environments [62, 63]. The maze consisted of three identical arms (34 × 5 × 20 cm). The task was conducted as described by Chiang et al. [59], with each mouse placed in the central area and allowed to explore freely for 8 min. The sequence and number of arm entries were recorded. Spontaneous alternation rate was calculated using the formula $\left(\frac{Number\;of\;unique\;triplet\;arm\;entries}{Total\;number\;of\;arm\;entries\;-\;2}\times100\%\right)$ serving as an indicator of the mouse’s short-term memory performance.

### Barnes Maze (BM)

The BM was utilized to evaluate spatial learning and long-term memory, capitalizing on mice’s aversion to bright and open spaces [64, 65]. The maze consisted of a white circular platform (92 cm diameter, 100 cm elevated) with 20 equally spaced holes (5 cm diameter) around the perimeter. A hole was randomly selected to be the escape hole, beneath which a dark escape box (17.5 × 11 × 3 cm) was placed. The platform was divided into four quadrants, with distinct visual cues surrounding the maze for orientation. The experiment was performed in two phases: training (learning) and probe (memory retrieval). During the training phase (days 1–5, 1 trial/day), each mouse was trained to locate the escape hole. Each trial began with the mouse being placed inside a white box (12.5 × 12.5 × 5 cm) at the center of the maze for 30 s to prevent directional bias. The box was then lifted, allowing the mouse to explore the maze for up to 4 min. If the mouse failed to locate the escape box within the time limit, it was gently guided to the target hole and placed inside the escape box for 30 s to reinforce learning. For the probe phase (days 6 and 9), the escape box was removed. The time that the mouse spent in the target quadrant within 4 min was recorded as an indicator of long-term memory retrieval.

### Histopathology and Serology Analysis

Liver and kidney tissues were isolated from sacrificed mice and fixed in 4% PFA (Sigma-Aldrich, USA). Blood samples were collected via submandibular vein puncture using microtainers (Becton Dickinson, USA). The blood was centrifuged at 13,000 × *g* for 15 min to isolate serum, which was then stored at − 80 °C. Liver and kidney tissues were embedded in paraffin, sectioned, and stained with hematoxylin and eosin (H&E) for the evaluation of inflammation and pathological lesions. All samples were sent to the National Animal Research Center for biochemical and pathological assessment. Serum biochemical parameters, including aspartate aminotransferase (AST), alanine aminotransferase (ALT), blood urea nitrogen (BUN), and serum creatinine (CREA), were measured.

### RT-qPCR

Total RNA was isolated from the hippocampi of mice or HT-22 cells using RNAzol (Molecular Research Center, USA). The RNA was then dissolved in nuclease-free water and stored at − 80 °C. RNA concentration was measured using a NanoDrop spectrophotometer (Thermo Fisher, USA). Complementary DNA (cDNA) was synthesized using the RevertAid First Strand cDNA Synthesis kit (Thermo Fisher, USA), with the following thermal protocol: 25 °C for 5 min, 42 °C for 60 min, and 70 °C for 5 min. Quantitative PCR was conducted using the StepOne Real-Time PCR System (Applied Biosystems, USA) in a total volume of 20 µL containing specific primers (100 nM) (Table 1), cDNA (100 ng), PowerTrack SYBR Green Master Mix (Thermo Fisher, USA), and nuclease-free water. The thermal cycling conditions were initially heated at 95 °C for 2 min, followed by 40 cycles of 95 °C for 5 s and 60 °C for 25 s. Melting curve analysis was then performed one cycle with 95 °C for 15 s, 60 °C for 1 min, and 95 °C for 15 s. Data were analyzed using StepOne software (Applied Biosystems, USA) and *Gapdh* was used as an internal control for normalization.

**Table 1:** Primers used in this study

Locus		Primer sequence	Product size (bp)
*SOD1*	Forward	AACCAGTTGTGTTGTCAGGAC	139
	Reverse	CCACCATGTTTCTTAGAGTGAGG	
*SOD2*	Forward	CAGACCTGCCTTACGACTATGG	113
	Reverse	CTCGGTGGCGTTGAGATTGTT	
*Catalase*	Forward	GGAGGCGGGAACCCAATAG	104
	Reverse	GTGTGCCATCTCGTCAGTGAA	
*GPx-4*	Forward	CCTCCCCAGTACTGCAACAG	147
	Reverse	GCACACGAAACCCCTGTACT	
*HO-1*	Forward	GCCTCCAGAGTTTCCGCATA	275
	Reverse	AGGAAGCCATCACCAGCTTAAA	
*GSH-Px*	Forward	GTGCAATCAGTTCGGACACCA	77
	Reverse	CACCAGGTCGGACGTACTTG	
*BDNF*	Forward	CCTGCATCTGTTGGGGAG	175
	Reverse	GCCTTGTCCGTGGACGTTT	
*IL-1β*	Forward	GAATGCCACCTTTTGACAGTG	119
	Reverse	TGGATGCTCTCATCAGGACAG	
*IL-6*	Forward	CCGGAGAGGAGACTTCACAG	129
	Reverse	TTGCCATTGCACAACTCTTT	
*IL-10*	Forward	AAGGCCATGAATGAATTTGA	198
	Reverse	TTCGGAGAGAGGTACAAACG	
*iNOS*	Forward	GTTCTCAGCCCAACAATACAAGA	127
	Reverse	GTGGACGGGTCGATGTCAC	
*Bax*	Forward	GATCCAAGACCAGGGTGGCT	198
	Reverse	CCTTCCCCATTCATCCCAG	
*Bcl-2*	Forward	GAACTGGGGGAGGATTGTGG	241
	Reverse	GCTGAGCAGGGTCTTCAGAG	
*Gapdh*	Forward	CATCACTGCCACCCAGAAGACTG	153
	Reverse	ATGCCAGTGAGCTTCCCGTTCAG	

### Western Blot (WB)

Each mouse hippocampal tissue was homogenized using lysis buffer [50 mM Tris–HCl (pH 7.4), 150 mM NaCl, 1% NP40 and 5 mM EDTA], supplemented with protease (Thermo Fisher, USA) and phosphatase inhibitors (Sigma-Aldrich, USA). The homogenized tissue was centrifuged at 13,200 × *g* for 15 min at 4 °C, and the supernatant was collected. Protein concentration was measured using the Pierce BCA Protein assay kit (Thermo Fisher, USA). For WB, 25 µg of protein was separated by SDS-PAGE and transferred to PVDF membranes (Millipore, Germany). Membranes were blocked with 5% skim milk in Tris-buffered saline with 0.1% Tween-20 (TBST) for 2 h at room temperature, followed by incubation with primary antibodies (Table 2) overnight at 4 °C. After washing with TBST, membranes were incubated with HRP-conjugated secondary antibodies (Table 2) for 1 h. Target protein signals were developed with ECL detection reagents (Millipore, Germany) and observed using the E-blot Touch Imager (e-Blot Photoelectric Technology, China). Quantification was performed with Image Studio Lite Ver 5.2 software (LICOR Biosciences, USA), and Gapdh was used as an internal loading control.

**Table 2:** Antibodies used in this study

Antibodies	Manufacturer	Catalog	Titer of IHC or WB
Primary antibodies			
NeuN	Millpore	MAB377	1:500 (IHC)
6E10	Biolegend	803001	1:500 (IHC)
GFAP	GeneTex	GTX108711	1:500 (IHC)
Iba-1	FUJIFILM Wako	019–19741	1:500 (IHC)
Phospho-Akt (Ser473)	Cell Signaling	4060S	1:1000 (WB)
Akt	Cell Signaling	9272S	1:1000 (WB)
NRF2	GeneTex	GTX103322	1:1000 (WB)
IDE	Abcam	ab32216	1:1000 (WB)
NF-κB p65	Cell Signaling	8242S	1:1000 (WB)
Phospho-p65 (Ser536)	GeneTex	GTX133899	1:1000 (WB)
SOD1	Santa cruz	sc-11407	1:1000 (WB)
SOD2	Millpore	06–984	1:1000 (WB)
PSD95	Cell Signaling	2507S	1:1000 (WB)
Synaptophysin	Abcam	ab14692	1:1000 (WB)
BDNF	Abcam	ab108319	1:1000 (WB)
GSK3β	Cell Signaling	9315S	1:1000 (WB)
Phospho-GSK3β (Ser9)	Cell Signaling	9336S	1:1000 (WB)
Bace1	Millpore	MAB5308	1:1000 (WB)
Bax	BD	556,467	1:1000 (WB)
Bcl-2	Santa cruz	ac-7382	1:1000 (WB)
Caspase-3	Cell Signaling	9662S	1:1000 (WB)
Beta-amyloid	Invitrogen	700254	1:500 (WB)
GAPDH	Arigo	ARG66330	1:2000 (WB)
Secondary antibodies			
Anti-mouse IgG, HRP-linked	Cell Signaling	7076S	1:2000 (WB)
Anti-rabbit IgG, HRP-linked	Cell Signaling	7074S	1:2000 (WB)
Goat anti-rabbit IgG H&L, Biotinylated	Vector Laboratories	BA-1000	1:500 (IHC)
Goat anti-mouse IgG H&L, biotinylated	Abcam	ab6788	1:500 (IHC)
Goat anti-rabbit IgG H&L (Alexa Fluor® 488)	Abcam	ab150077	1:500 (IHC)

### Immunohistochemistry (IHC) Staining

After anesthetizing with 0.4 g/kg Avertin (Sigma-Aldrich, USA), mouse perfusion was performed to remove the blood, followed by brain collection, dehydration, and sectioning. The brain sections were rinsed with PBS solution three times and then treated with 3% hydrogen peroxide (Sigma-Aldrich, USA) at room temperature for 30 min. The sections were blocked using 2% normal horse serum in PBS for 1 h at 37 °C. Primary antibodies (Table 2) were then applied to the sections at room temperature overnight, followed by secondary antibodies (Table 2) at room temperature for 1 h, and then incubated with Avidin–biotin complex (Vector Laboratories, USA) for 1 h. Sections were stained with DAB substrate kits (Vector Laboratories, USA), mounted onto slides, visualized using a microscope (Leica SP2, Germany) and quantified using ImageJ software (National Institutes of Health, USA).

### Statistical Analysis

All experimental data were obtained from at least three independent experiments and expressed as the mean ± standard error of the mean (SEM). Statistical analysis was performed using one-way ANOVA followed by Fisher’s Least Significant Difference (LSD) tests for multiple comparisons. A *p*-value < 0.05 was considered statistically significant.

## Results

### Effects of FS on Aβ-Induced HT-22 Neurotoxicity

**Fig. 1:**
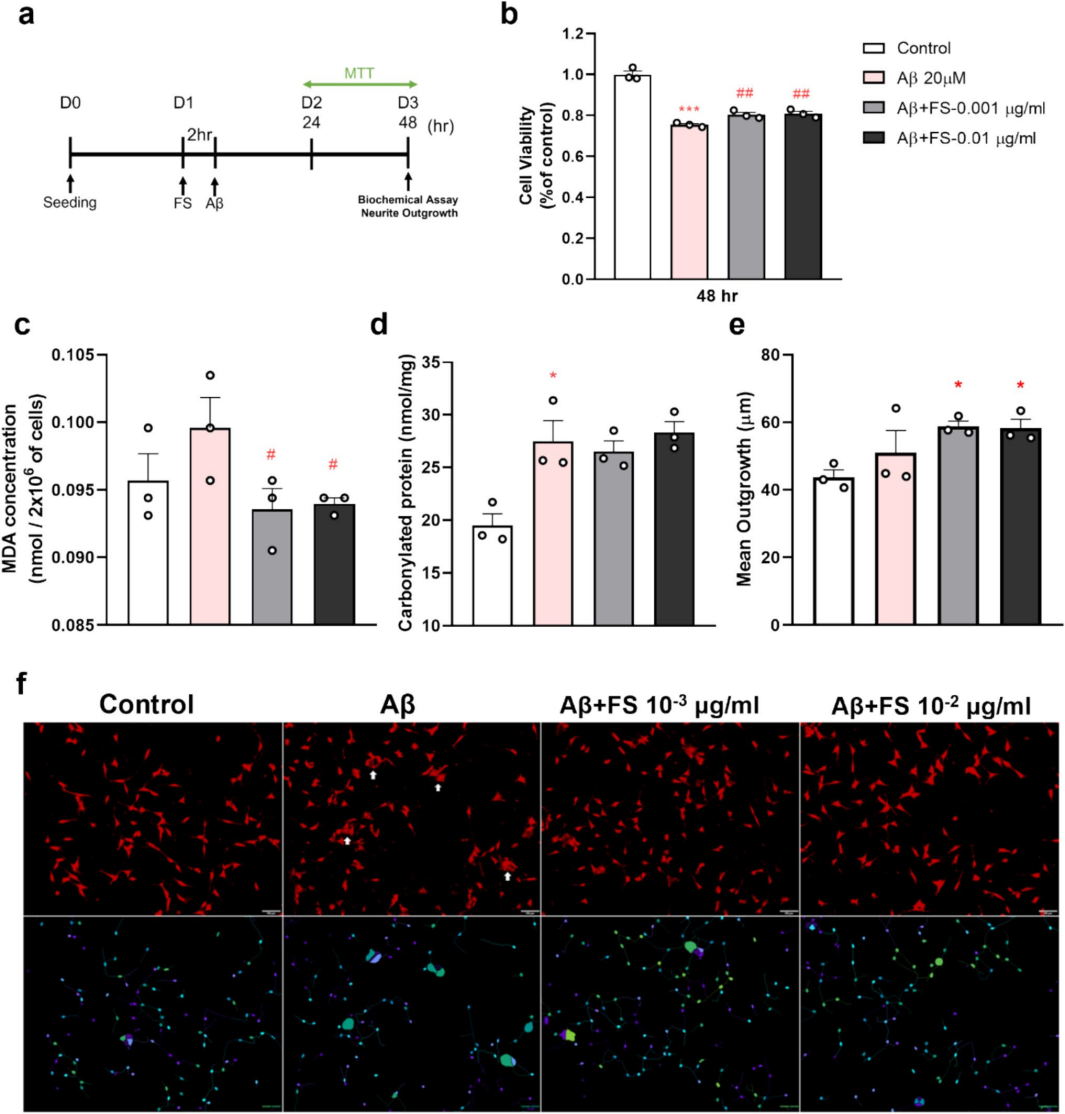
Effects of FS on in vitro HT-22 cell AD model under Aβ-induced neurotoxicity. **a** The experimental flow chart. **b** Cells were pretreated with FS for 2 h and then co-incubated with 20 μM Aβ for 48 h to assess viability using MTT assay. **c** Results of lipid oxidation stress analysis. **d** Results of protein oxidation stress analysis. **e** Results of neurite outgrowth quantitative. **f** Representative photographs of neurite outgrowth staining observed by microscope. White arrows point to cells with abnormal morphologies; *p < 0.05; ***p < 0.001 compared to the WT + S group; #*p* < 0.05; ##*p* < 0.01 compared to the TG + S group (*n* = 3)

We used the MTT assay to evaluate the FS cytotoxicity and its protective effects against Aβ-induced neurotoxicity in HT-22 cells, as shown in the diagram (Fig. 1a and Fig. S1a). After 48 h of treatment, FS at concentrations of 0.001 µg/ml and 0.01 µg/ml significantly improved HT-22 cell viability under 20 µM Aβ toxicity (Fig. S1b). These results suggest that FS may exert neuroprotective effects in this in vitro model (Fig. 1b). Additionally, a lipid peroxidation assay was performed to assess oxidative damage to cellular lipids. FS-treated groups (0.001 and 0.01 µg/ml) significantly reduced MDA levels compared to the Aβ-only group (Fig. 1c), indicating that FS effectively mitigated lipid oxidative stress induced by Aβ. In contrast, regarding protein oxidation, although the Aβ group showed significantly higher oxidative stress compared to the control, no significant difference was observed in the levels of protein oxidation between the FS-treated group and the Aβ group (Fig. 1d), suggesting that FS has a limited impact on protein oxidative damage. We also examined the effect of FS on mitochondrial activity with the NAD^+^/NADH ratio. Mitochondrial activity was markedly decreased in the Aβ group compared to the control group. However, FS treatment did not significantly improve the NAD⁺/NADH ratio (Fig. S1c), implying that FS may not influence mitochondrial function under these conditions.

### Effects of FS on Neurite Outgrowth in Aβ-Treated HT-22 Cell

We measured the neurite outgrowth of HT-22 cells to evaluate whether FS treatment exerts a neuronal protective effect. The mean neurite outgrowth in the two concentrations of FS-treated groups was significantly increased compared to the control group, whereas the TG + S group showed no significant difference from the control group (Fig. 1e and f). Morphological analysis revealed that Aβ (20 µM) treatment led to a higher proportion of cells with abnormal morphology, while FS-treated groups showed a notable reduction in the morphological deficit (Fig. 1f). These observations suggest that FS treatment may promote neurite outgrowth and improve neuroplasticity under Aβ damage.

### Evaluation of Toxicity of FS in Mice

To assess the toxic effect of FS in mice, body weight was monitored at five time points throughout the experiment. No significant differences were observed among the four groups (Fig. S2a). Serum biochemical analysis also showed no significant differences between groups (Fig. S2b-e). Histopathological examination of liver and kidney tissues revealed that all indicators in the FS-treated groups remained below moderate levels (Table S2). Interestingly, moderate glycogen accumulation observed in the TG + S group was reduced to a mild level following FS treatment. Additionally, the OFT conducted to assess motor activity showed no significant differences among the groups (Fig.  S2f). These findings suggest that a long-term administration of FS is well tolerated and does not induce significant toxicity in mice.

### Effects of FS on Spatial Cognitive Function in Mice

**Fig. 2:**
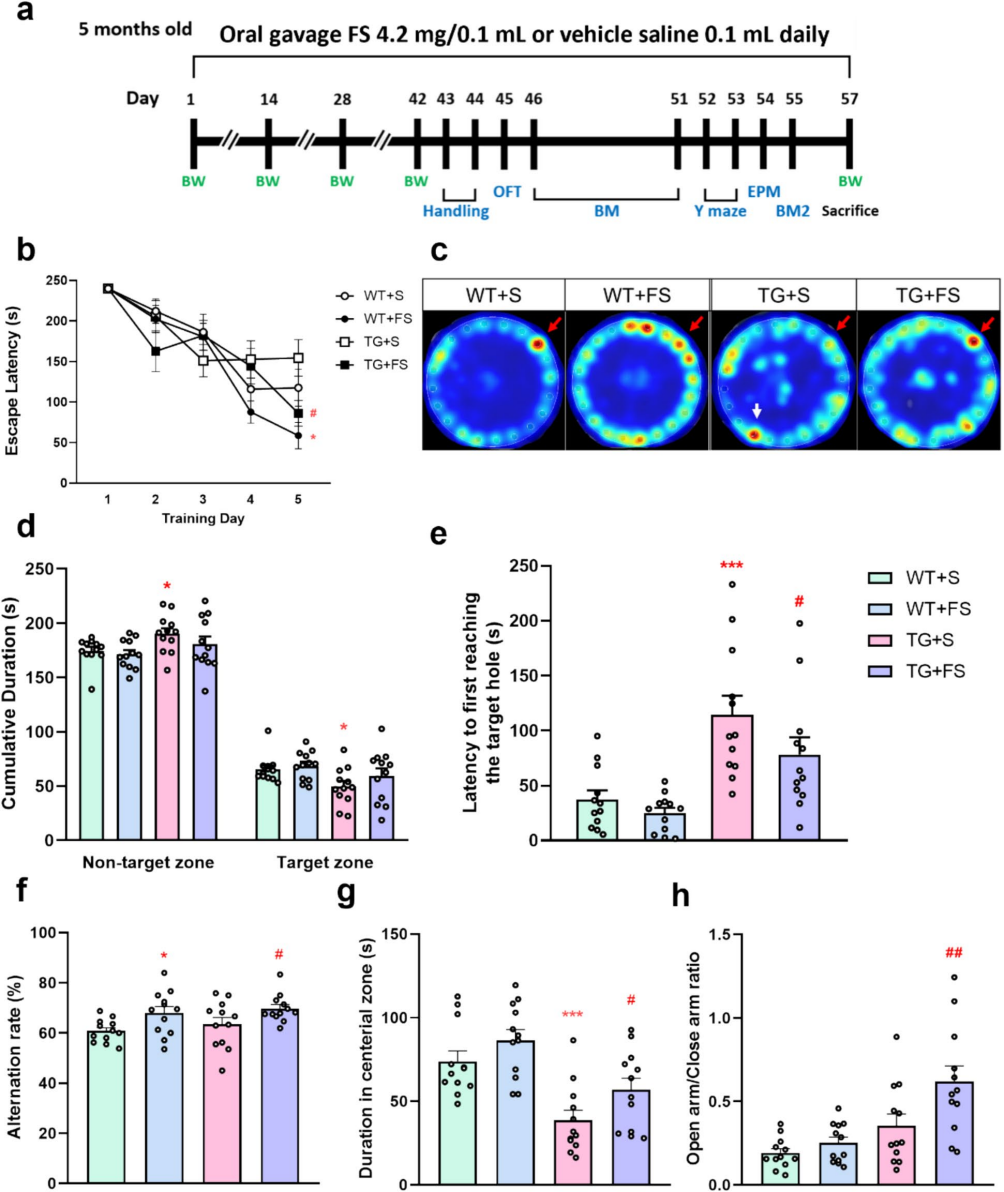
Effects of FS on mouse behavioral changes. **a** The timeline of the animal experimental process. **b** The time mice required to enter the escape box during the BM training phase. **c** Heatmap of mice location during the BM probe 2. Red arrows indicate the target, while the white arrow points to a highly intensive non-target area. **d** Time spent by mice in the target and non-target quadrants during the BM probe 2. **e** The latency to first reach the target hole. **f** The short-term memory determined by the spontaneous alternation ratio in the Y maze. **g** The time mice spent in the center zone of the OFT. **h** The open arm/closed arm ratio of EPM; *p < 0.05; **p < 0.01; ***p < 0.001 compared to the WT + S group; #*p* < 0.05; ##*p* < 0.01 compared to the TG + S group (*n* = 12)

The experimental design of the animal study is shown in Fig. 2a. During the training phase of BM, both WT and TG mice with FS treatment exhibited a significantly reduced latency to locate the escape box compared to their respective saline-treated controls on day 5 (Fig. 2b). Furthermore, the number of animals successfully entering the escape box in the FS-treated group was higher than in the saline-treated group (Fig. S3a). In the first minute of the BM probe 1, the TG + S group spent significantly less time in the target hole compared to the WT + S group, whereas the TG + FS group spent significantly more time, indicating improved memory retention (Fig. S3b). In both BM probes 1 and 2, the TG + S group spent significantly less time in the target zone compared to the WT + S group, and the FS treatment slightly increased the period (Fig. 2c and d and S3c, d). Moreover, the TG + S group exhibited a significantly longer latency to first reach the target hole compared to the WT + S group, and this delay was significantly reduced in the TG + FS group (Fig. 2e). These results suggest that FS administration improves the performance of long-term spatial memory in 5 × FAD mice.

### Effects of FS on Short-Term Memory and Anxiety Levels

The Y-maze test was performed to evaluate short-term memory. The spontaneous alternation rate of the WT + FS and TG + FS groups was significantly higher than their respective saline-treated controls, suggesting that FS treatment may enhance short-term memory (Fig. 2f). There were no significant differences found in the number of arm entries among the groups (Fig. S4), indicating comparable locomotor activity. The level of anxiety in mice was assessed using the OFT and EPM. In the OFT, the TG + S group spent significantly less time in the center area compared to the WT + S group, while FS treatment significantly increased center zone exploration (Fig. 2g). Similarly, in the EPM, the TG + FS group spent significantly longer time in the open arms compared to the TG + S group (Fig. 2h). Together, these results suggest that FS treatment may alleviate anxiety-like behavior in TG mice.

### Effects of FS on the Amyloid Plaque Accumulation in Mouse Hippocampus

**Fig. 3:**
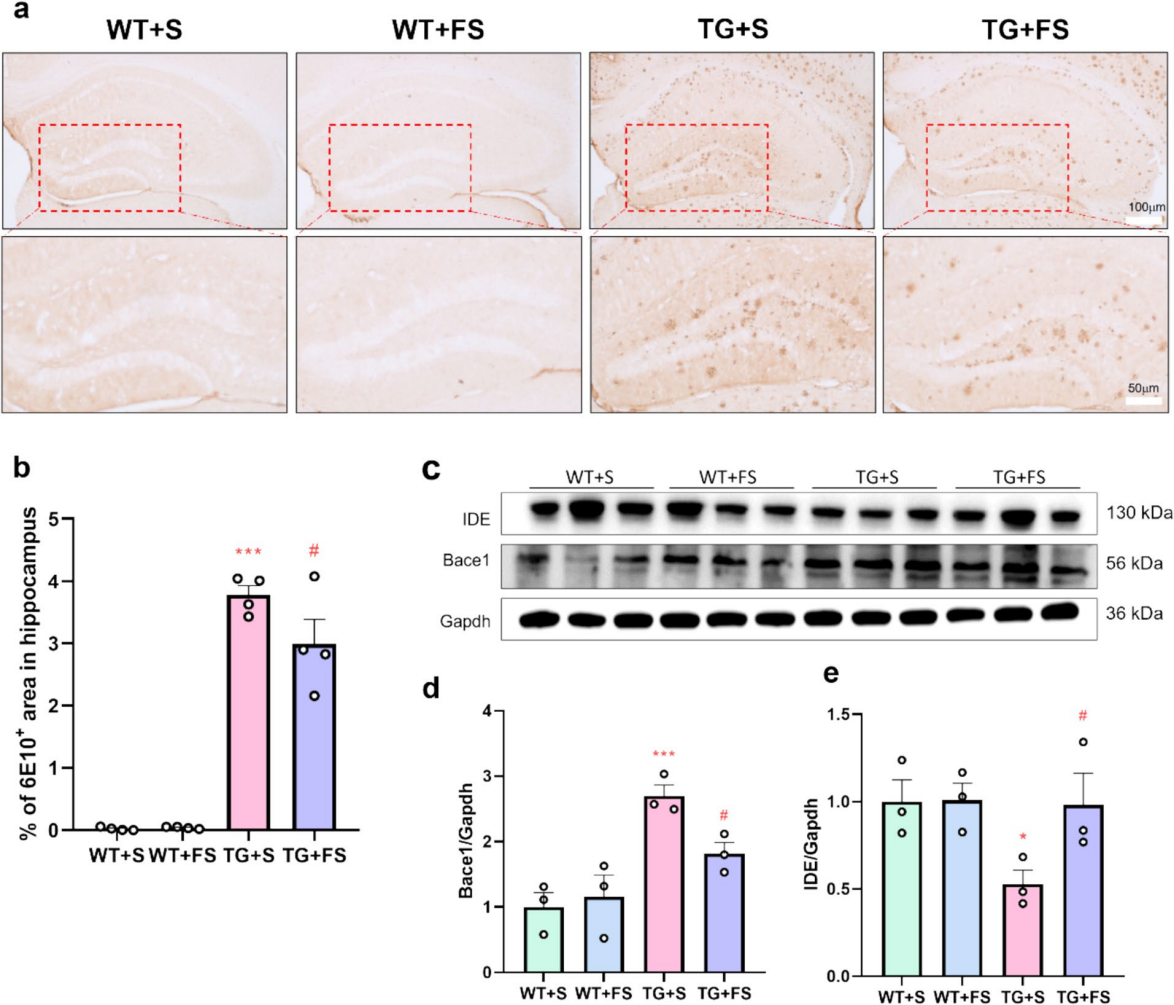
FS treatment ameliorates Aβ pathology in the hippocampi of 5 × FAD mice. **a** Representative IHC analysis using 6E10 antibody in the hippocampus. **b** Quantification of the 6E10.^+^ area in hippocampi of mice (*n* = 4). **c** WB of IDE and Bace1. Statistical analysis of Bace1 (**d**) and IDE (**e**); *p < 0.05; ***p < 0.001 compared to the WT + S group; #*p* < 0.05 compared to the TG + S group (*n* = 3)

Amyloid plaque accumulation is a hallmark of AD pathology, primarily resulting from an imbalance between Aβ production and clearance [66, 67]. IHC staining revealed that the TG + S group exhibited a significant amyloid plaque burden in the hippocampus compared to the WT + S group, whereas FS treatment markedly reduced plaque accumulation (Fig. 3a and b). To investigate whether FS modulates amyloid pathology through the regulation of Aβ production and clearance, we assessed the expression of β-secretase (Bace1) and insulin-degrading enzyme (IDE) in the hippocampus of 5 × FAD mice. WB analysis showed Bace1 expression was significantly increased in the TG + S group compared to the WT + S group. FS treatment significantly reduced Bace1 expression in the TG mice (Fig. 3c and d). In contrast, IDE expression was significantly reduced in the TG + S group compared to the WT + S group. FS treatment significantly restored IDE expression levels in the TG mice (Fig. 3c and e). Moreover, FS treatment significantly reduced soluble Aβ oligomers in the hippocampi of 5 × FAD mice (Fig. S5a-c). These findings suggest that FS reducing the amyloid plaque burden could be mediated by inhibiting Bace1 expression to reduce Aβ production and by promoting IDE expression to enhance Aβ clearance.

### Effects of FS on Synaptic and BDNF Proteins in Mouse Hippocampus

**Fig. 4:**
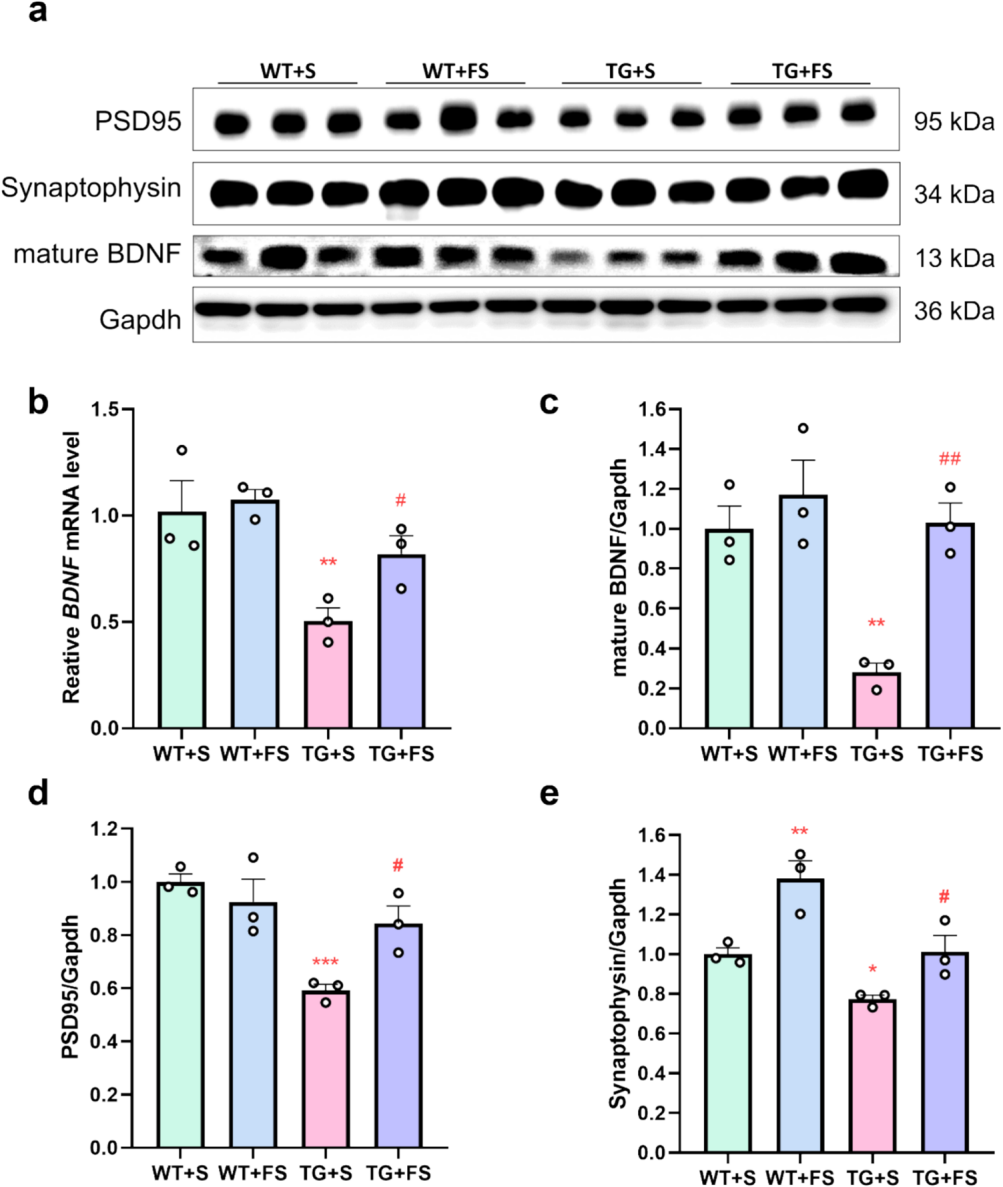
FS enhances synaptic-associated protein and BDNF expression in hippocampi of 5 × FAD mice. **a** Results of WB analyses of PSD95, synaptophysin, mature BDNF, and Gapdh. **b** The mRNA level of *BDNF* in the mouse hippocampus. Statistical analysis of WB results of mature BDNF (**c**), PSD95 (**d**), and synaptophysin (**e**); *p < 0.05; **p < 0.01; ***p < 0.001 compared to the WT + S group; #*p* < 0.05; ##*p* < 0.01 compared to the TG + S group (*n* = 3)

We investigated whether FS treatment activates BDNF signaling in the hippocampus and promotes the expression of synaptic plasticity-related proteins in mice. Both WB and qPCR analyses showed that BDNF levels were significantly reduced in the TG + S group compared to the WT + S group, whereas FS treatment significantly restored BDNF expression in the TG + FS group (Fig. 4a–c). In addition, WB results showed that the levels of PSD95 and synaptophysin were decreased in the TG + S group but were significantly restored following FS treatment (Fig. 4a, d, and e). These findings suggest that FS may modulate BDNF expression and thereby enhance synaptic plasticity in the hippocampus of 5 × FAD mice.

### Effects of FS on the NF-κB Signaling and Gliosis in Mouse Hippocampus

**Fig. 5:**
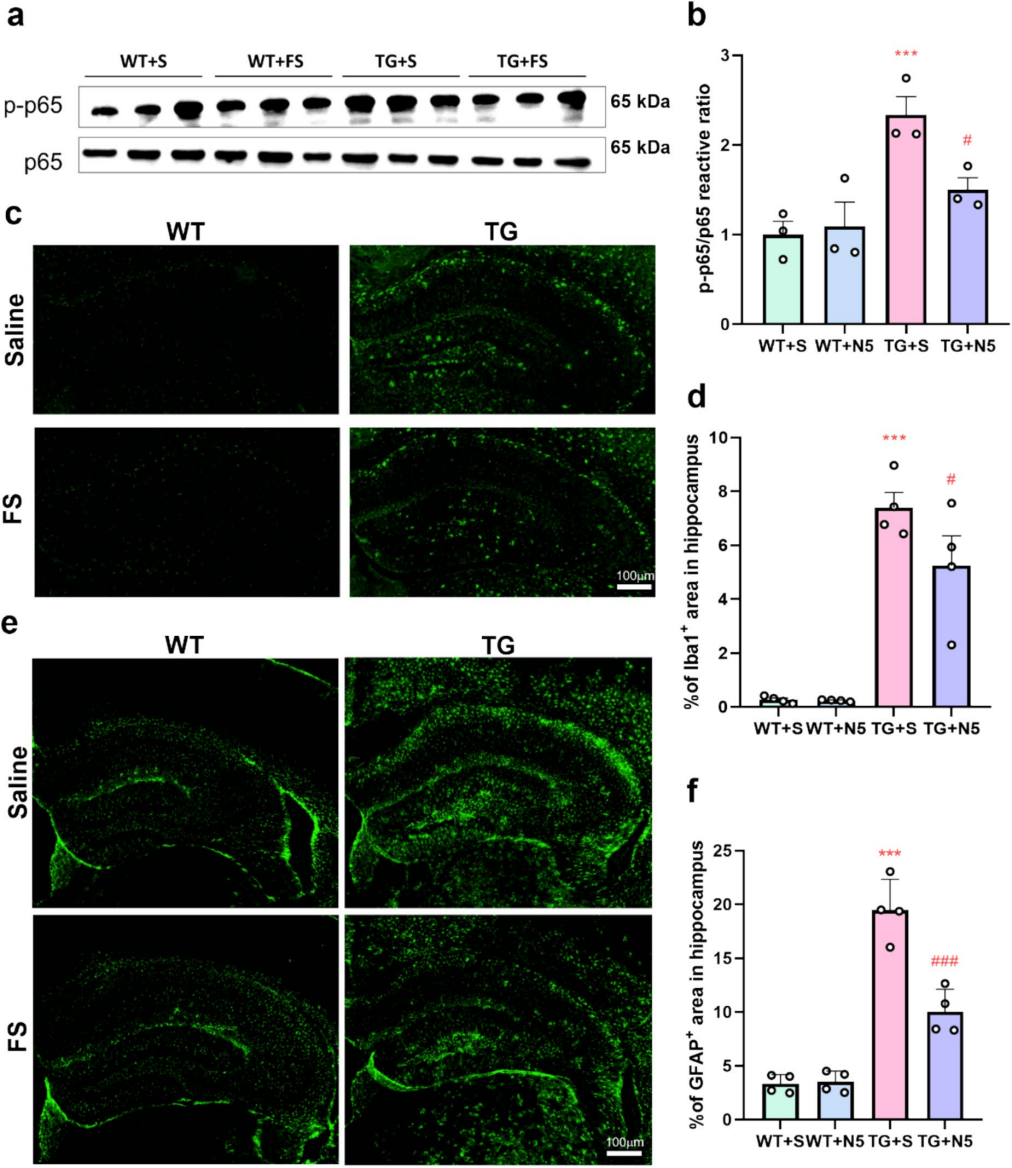
FS inhibits the NF-κB signaling and gliosis in the hippocampi of 5 × FAD mice. **a** Expressions of p-p65 and p65 in the hippocampi of mice were analyzed by WB. **b** Statistical analysis of p-p65/p65 ratio of the 4 groups. **c** Immunoreactivity of Iba-1 in the hippocampus. **d** Quantification of Iba-1^+^ area in the hippocampus (*n* = 4). **e** Immunoreactivity of GFAP in the hippocampus. **f** Quantification of GFAP^+^ area in the hippocampus (*n* = 4); ***p < 0.001 compared to the WT + S group; #*p* < 0.05; ###*p* < 0.001 compared to the TG + S group

NF-κB is a transcription factor composed of p50 and p65 subunits that regulates the expression of various pro-inflammatory cytokines and *iNOS* genes [68, 69]. To investigate whether the protective effects of FS involve anti-neuroinflammatory mechanisms, we examined the NF-κB pathway and inflammation-related markers in the hippocampus. WB analysis revealed that the TG + S group exhibited significantly elevated phosphorylated p65 (p-p65) compared to the WT + S group. FS treatment significantly reduced p-p65 expression in TG mice (Fig. 5a and b). A similar trend was observed for iNOS expression, which was significantly downregulated following FS treatment (Fig. S6a). However, the increased pro-inflammatory *IL-1β* and *IL-6*, and the reduced anti-inflammatory cytokines *IL-10* gene expression in the TG + S group were not significantly altered by the administration of FS (Fig. S6b-d). Immunohistochemical staining for neuroinflammation markers, including Iba-1 (a microglial marker) and GFAP (an astrocyte marker) showed that gliosis was significantly increased in the TG + S group compared to the WT + S group. FS treatment significantly mitigated the levels of both Iba-1 and GFAP, suggesting that FS effectively alleviates neuroinflammation in 5 × FAD mice (Fig. 5c–f). These results suggest that FS treatment inhibits NF-κB signaling and alleviates gliosis in the hippocampus of 5 × FAD mice.

### Effects of FS on the Neuronal Loss in Mouse Hippocampus

**Fig. 6:**
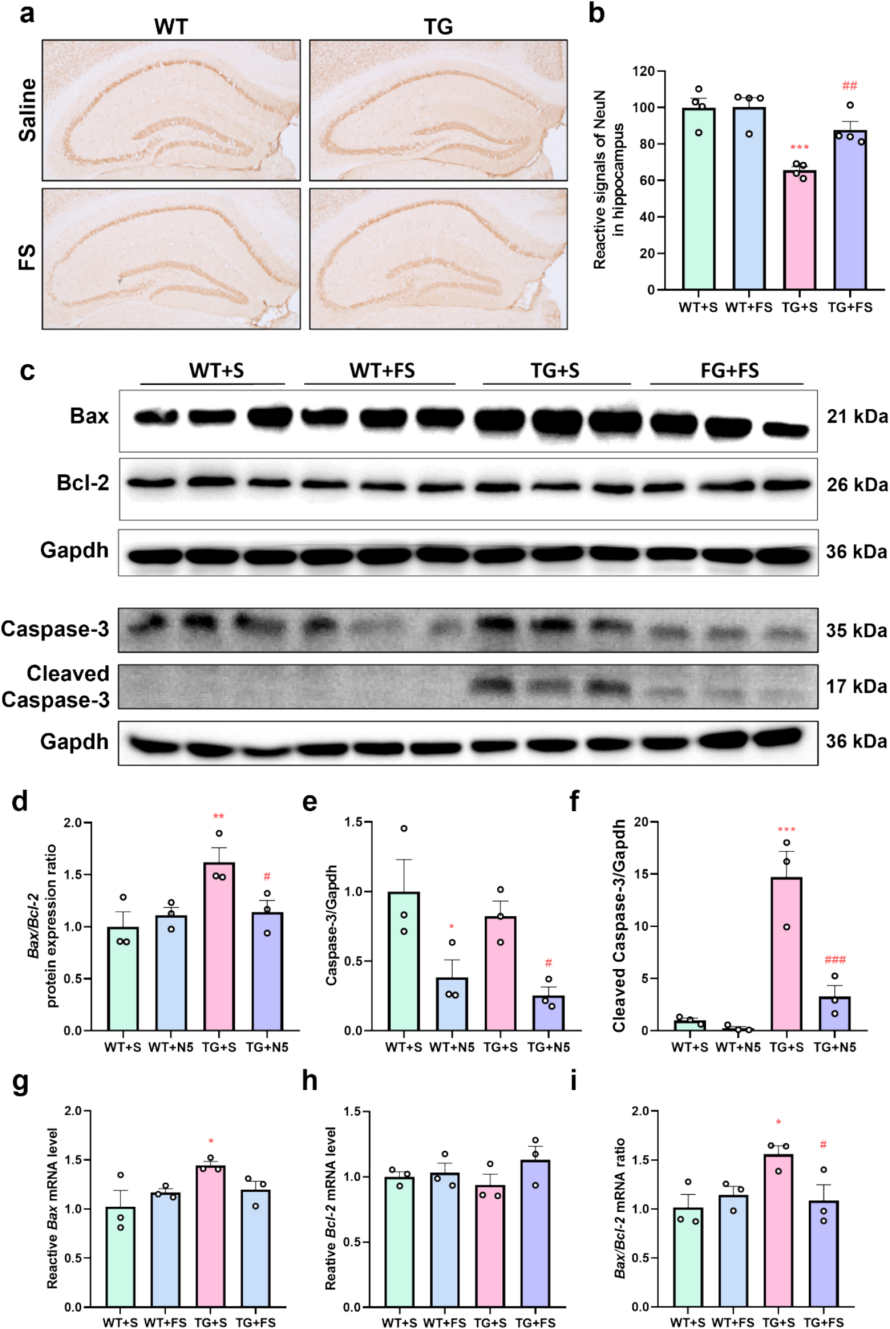
FS treatment ameliorates neuronal loss in 5 × FAD mouse hippocampus. **a** IHC analysis of NeuN in hippocampal neurons. **b** Quantification of the optical density of NeuN.^+^. **c** Immunoblots of Bax, Bcl-2, and Caspase-3 in hippocampal tissues of mice. Statistical analysis of Bax/Bcl-2 ratio (**d**), Caspase-3 (**e**), and Cleaved-Caspase-3 (**f**) from the WB. The mRNA expression of *Bax* (**g**), *Bcl-2* (**h**), and *Bax/Bcl-2* ratio (**i**) in hippocampi of mice; *p < 0.05; **p < 0.01; ***p < 0.001 compared to the WT + S group; #*p* < 0.05; ##*p* < 0.01 compared to the TG + S group (*n* = 3)

IHC analysis was performed to evaluate NeuN expression in the hippocampus. Compared to the WT + S group, NeuN expression was reduced in the TG + S group but significantly restored in the TG + FS group (Fig. 6a and b). Furthermore, the TG + FS group showed decreased apoptosis-related indicators, including Bax, Cleaved-Caspase-3, as well as the Bax/Bcl-2 ratio compared to the TG + S group (Fig. 6c–i). These findings suggest that FS treatment may mitigate neuronal apoptosis and help preserve neuronal survival in 5 × FAD mice.

### Effects of FS on the Akt/GSK3β/Nrf2 Pathway in Mouse Hippocampus

**Fig. 7:**
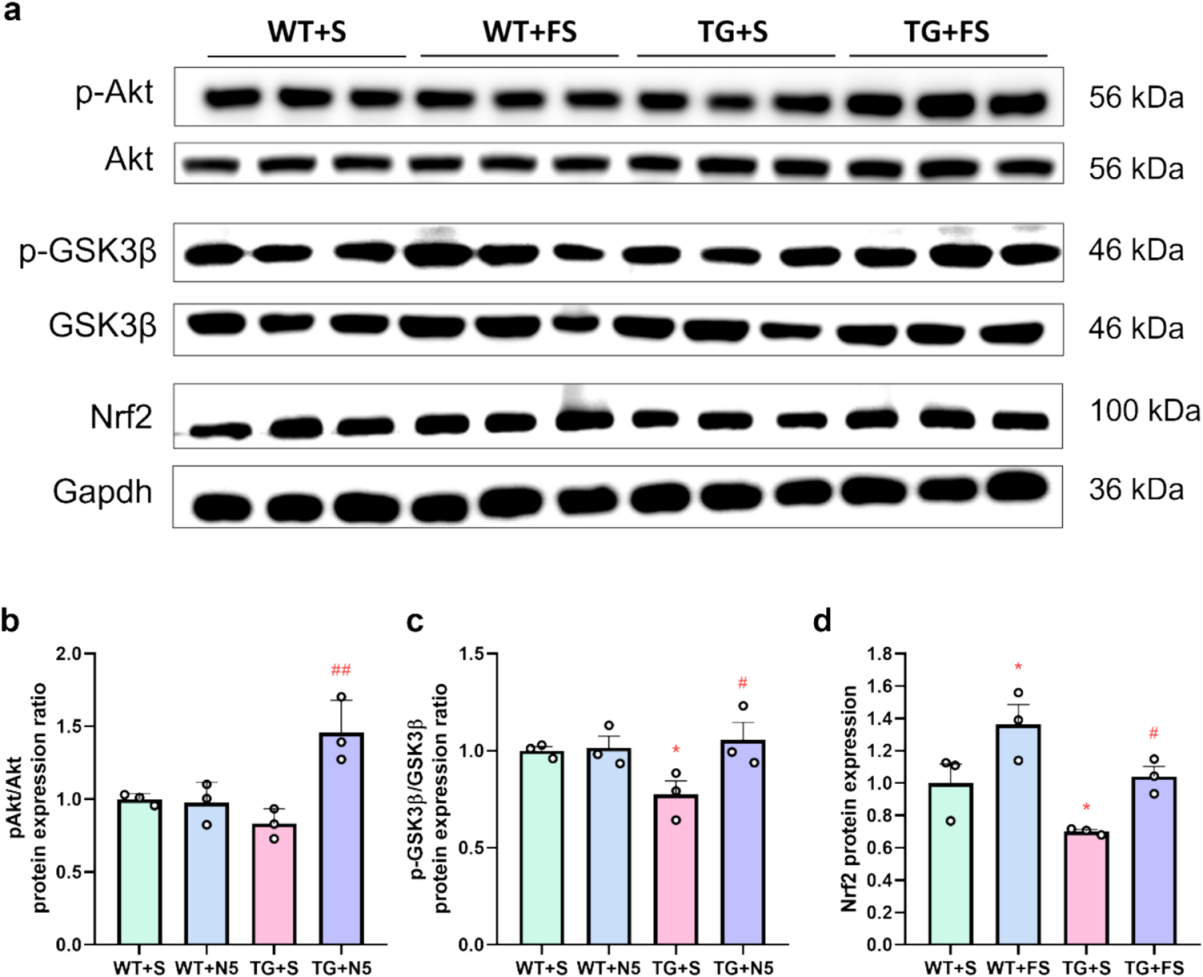
FS rescues the inactivation of the Akt/GSK3β/Nrf2 pathway in the 5 × FAD hippocampus. **a** Results of WB analysis of p-Akt, p-GSK3β, and Nrf2. Statistical analysis of p-Akt (**b**), p-GSK3β (**c**), and Nrf2 (**d**); *p < 0.05 compared to the WT + S group; #*p* < 0.05; ##*p* < 0.01 compared to the TG + S group (*n* = 3)

The PI3K/Akt signaling pathway plays a critical role in regulating cell metabolism, survival, and apoptosis, and has been implicated in the pathogenesis of AD [70, 71]. Activation of this pathway influences various downstream processes, with glycogen synthase kinase 3β (GSK3β) serving as a key regulator [72, 73]. Inhibition of GSK3β has been reported to enhance nuclear factor erythroid 2-related factor 2 (Nrf2) activity and modulate NF-κB signaling [74, 75]. Therefore, we investigated whether FS modulates the Akt/GSK3β/Nrf2 pathway to promote antioxidant enzyme expression and inhibit NF-κB-mediated neuroinflammation. WB analysis showed that the level of phosphorylated Akt (p-Akt) was significantly increased in the TG + FS group compared to the TG + S group (Fig. 7a and b). Additionally, in the TG + S group, p-GSK3β and Nrf2 expression levels were significantly lower than those in the WT + S group (Fig. 7a, c, and d), suggesting that FS activated the Akt/GSK3β/Nrf2 pathway in the hippocampi of 5 × FAD mice.

### Effects of FS on the Expression of Antioxidant Enzymes in Mouse Hippocampus

**Fig. 8:**
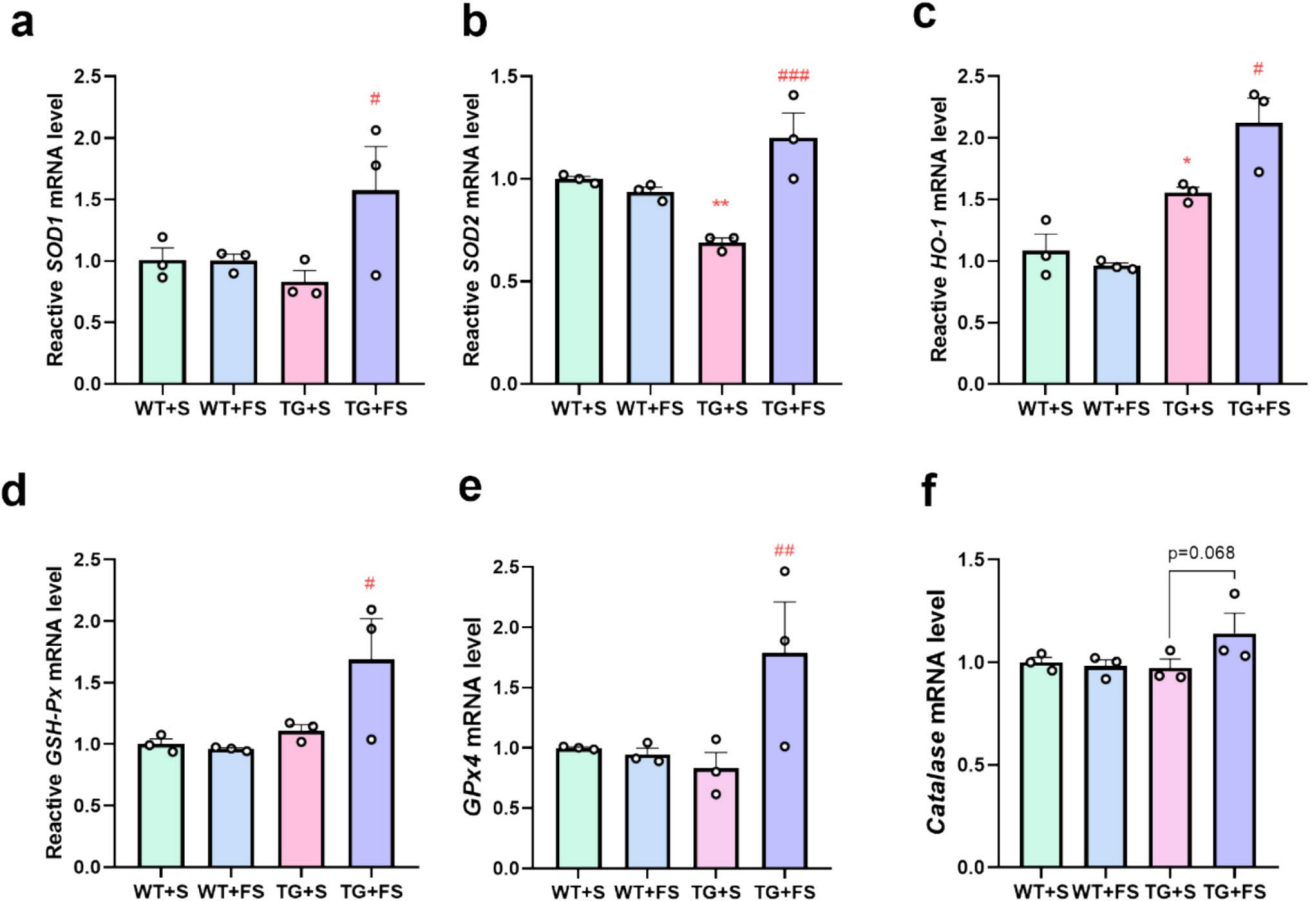
FS increases the gene expression of antioxidant enzymes in 5 × FAD hippocampus. The relative mRNA expression levels of *SOD1 *(**a**), *SOD2 *(**b**), *HO-1 *(**c**), *GSH-Px *(**d**), *GPx4 *(**e**), and *Catalase *(**f**); *p < 0.05 compared to the WT + S group; #*p* < 0.05; ##*p* < 0.01; ###*p* < 0.01 compared to the TG + S group (*n* = 3)

Aβ accumulation is known to induce oxidative stress in the AD brain [76, 77]. To investigate the antioxidant effects of FS and its potential involvement in Nrf2 pathway activation, we examined the expression of several antioxidant enzymes’ mRNA levels in the hippocampus of mice. In TG + FS mice, FS treatment significantly upregulated the gene expression levels of *SOD1* (Fig. 8a), *SOD2* (Fig. 8b), *HO-1* (Fig. 8c), *GSH-Px* (Fig. 8d), and *GPx4* (Fig. 8e), but had no significant effect on *Catalase* expression (Fig. 8f). In addition, WB analysis showed that SOD1 expression was significantly elevated in both the WT-FS and TG-FS groups compared to their respective saline-treated controls (Fig. S7a, b). Although not statistically significant, SOD2 expression exhibited a similar upward trend in FS-treated groups (Fig. S7a, c).

## Discussion

AD is the most common neurodegenerative disease, characterized by progressive cognitive decline, synaptic dysfunction, and Aβ plaque accumulation in the brain [78]. It has become an increasingly severe global public health challenge, imposing significant economic and social burdens [79]. Although the U.S. Food and Drug Administration recently approved AD drugs show potential in improving disease outcomes, but the long-term efficacy and safety of these drugs still require further validation. Therefore, the search for safe and effective preventive or therapeutic strategies for AD remains an urgent priority. Research has shown that diet plays a crucial role in modulating the risk and progression of neurodegenerative diseases, including AD [80, 81]. Soybeans and their derived components, such as isoflavones and phytosterols, have been reported to reduce oxidative stress, attenuate inflammation, and improve cognitive functions [82, 83]. This study investigates the potential preventive/therapeutic effects of FS, a fermented soybean pulp, on the progression of AD pathologies in 5 × FAD mice. Research focused on safety and its underlying mechanisms in reducing amyloid plaque accumulation, alleviating neuroinflammation, and enhancing antioxidant defenses.

First, no significant differences were observed in mouse body weight, serum biochemical parameters, liver and kidney histology, or motor activity in the OFT. These findings suggest that long-term administration of FS is safe. An interesting finding was the moderate glycogen accumulation in the livers of 5 × FAD mice reduced by FS treatment. It was reported that AD is associated with metabolic dysfunction, including insulin resistance and impaired glucose regulation, which can affect hepatic glycogen storage [84]. Disruptions in glycogen metabolism may contribute to brain energy deficits seen in AD. We hypothesize that the reduction in hepatic glycogen storage following FS treatment suggests improved glycogen metabolism, leading to increased glucose availability to support the brain’s high energy demands and may repair AD pathology.

The 5 × FAD model shows early Aβ deposition, gliosis, and synaptic dysfunction [85], making it a widely used model in AD research [85, 86]. FS significantly reduced amyloid plaque accumulation in the hippocampus. Mechanistically, FS reduced the expression of BACE1, a key enzyme in Aβ production, and promoted the expression of IDE, an Aβ degradation enzyme, suggesting FS’s dual action in reducing Aβ accumulation. In addition, Aβ deposition induces oxidative stress, which plays a crucial role in AD pathogenesis [76, 87]. FS treatment upregulated expression levels of antioxidant enzymes, including SOD1, SOD2, HO-1, and GSH-Px, in the hippocampus of 5 × FAD mice. However, no significant upregulation was observed in WT + FS mice. This may be due to the intrinsic lower oxidative stress levels in wild-type mice [88, 89].

The NF-κB signaling pathway is a pivotal regulator of many pathways, including neuroinflammation, activation of microglia, oxidative stress-related complications, and apoptosis, all of which have significant implications in AD [90–92]. FS treatment reduced p-p65 levels and downregulated *iNOS* mRNA expression in the hippocampus. IHC analysis showed that FS significantly suppressed gliosis, indicated by reducing the levels of Iba-1 and GFAP. These results suggest that FS mitigates AD-associated neuroinflammation, at least in part, via inhibition of NF-κB signaling. Furthermore, FS treatment rescued the expression of synaptic proteins, PSD95 and synaptophysin, and restored the levels of mature BDNF, which are essential for synaptic plasticity [93]. FS likely exerts its effects by modulating the BDNF-associated AKT/GSK3β/NRF2 pathway [94, 95], as evidenced by increased levels of pAKT/AKT, pGSK3β/GSK3β, and NRF2. This pathway not only promotes antioxidant defense but also suppresses NF-κB-mediated inflammation and Bace1 expression [96–98], suggesting that FS achieves its neuroprotective effects by targeting coordinated mechanisms involved in AD pathogenesis. Furthermore, FS contains several bioactive components, including genistein and GABA, which have been individually associated with neuroprotective effects [99–102]. In addition to these identified compounds, we cannot exclude the possibility that other components within FS may exert beneficial effects through microbial metabolism or physiological regulation, which could indirectly impact brain function. This is especially relevant considering the complex interaction between diet, gut microbiota, and the central nervous system.

In the animal behavioral experiments, FS improved both short- and long-term memories, as evidenced by increased spontaneous alternation rate in the Y maze and reduced the latency to locate the escape box during BM tests. Additionally, FS reduced anxiety-like behaviors, as shown by increased exploration time in the open arms of the EPM and the central area of the OFT. These findings highlight FS’s potential to counteract the cognitive and emotional impairments associated with AD.

## Conclusion

Our findings demonstrate that FS treatment effectively alleviates AD-associated pathologies in 5 × FAD mice by targeting several key mechanisms, including Aβ metabolism, synaptic plasticity, oxidative stress, and neuroinflammation. The multi-targeted actions of FS, particularly its modulation of the Akt/GSK3β/Nrf2 pathway, support its potential as a promising therapeutic strategy for AD. Furthermore, this study highlights the current environmental awareness; the repurposing of soybean pulp not only enhances its potential application in neurodegenerative disease prevention but also promotes sustainable green recycling.

## Supplementary Information

Below is the link to the electronic supplementary material.

Supplementary Material 1 (DOCX 1.57 MB)

Supplementary Material 2 (DOCX 23.2 MB)

## Data Availability

No datasets were generated or analysed during the current study.

## Abbreviations

AD: Alzheimer’s disease
ACOC: Association of Official Analytical Chemists
Akt: Protein kinase B
APP: Amyloid precursor protein
Aβ: Amyloid beta
BACE1: β-Secretase
BBB: Blood-brain barrier
BDNF: Brain-derived neurotrophic factor
BM: Barnes maze
CAT: Catalase
CNS: Central nervous system
EPM: Elevated plus maze
FAD: Familial Alzheimer’s disease
FS: Fermented soybean pulp
GFAP: Glial fibrillary acidic protein
GPx4: Glutathione peroxidase 4
GSH-Px: Glutathione peroxidase
GSK3β: Glycogen synthase kinase 3β
HO-1: Heme oxygenase-1
Iba-1: Ionized calcium-binding adapter molecule 1
IDE: Insulin-degrading enzyme
IHC: Immunohistochemistry
IL-1β: Interleukin 1 beta
IL-6: Interleukin 6
iNOS: Inducible nitric oxide synthase enzyme
NeuN: Neuronal nuclei
NFT: Neurofibrillary tangles
NF-κB: Nuclear factor kappa-light-chain-enhancer of activated B cells
NRF2: Nuclear factor erythroid 2-related factor 2
OFT: Open field test
ROS: Reactive oxygen species
SOD1: Superoxide dismutase type 1
SOD2: Superoxide dismutase type 2
TNF-α: Tumor necrosis factor-α
WB: Western blot
