# Supplementary material for: Fermented Soybean Pulp Alleviates Disease Progression of 5×FAD Model Mice
Source: Mol Neurobiol. 2025 Jul 15;62(11):14121–39. doi: 10.1007/s12035-025-05191-y (PMC12511221; doi:10.1007/s12035-025-05191-y)
Supplement: Supplementary file 1 — Supplementary Material 1 (DOCX 1.57 MB) [file 12035_2025_5191_MOESM1_ESM.docx]

**Supplemental Table and Figures**

**Supplemental Table 1.** The potential bioactive components in FS

| **Composition Analysis** | **Pre-Fermented soybean** | **Fermented soybean (FS)** |
| --- | --- | --- |
| **Isoflavones (μg/g)** | | |
| **Daidzin** | 363.31±122.42 | 68.89±4.69 |
| **Glycitin** | 79.39±30.20 | 88.15±7.76 |
| **Genistin** | 538.73±177.04 | n.d |
| **Daidzein** | 96.72±31.27 | n.d |
| **Genistein** | 150.11±42.51 | 1123.26±96.48 |
| **Anthocyanidin (μg/g)** | | |
| **Delphinidin** | n.d | n.d |
| **Cyanidin** | n.d | n.d |
| **D3G** | n.d | n.d |
| **C3G** | 52.51±1.94 | 0.57±0.16 |
| **Cellulose content (%)** | 6.15±0.03 | 6.15±0.64 |
| **GABA (μg/g)** | n.d | 1576.1±2.7 |

**Supplemental Table 2.** Histopathological lesion severity scores of each group (n=3/group).

| Histopathological findings | **Animal Groups** | | | | | | | | | | | |
| --- | --- | --- | --- | --- | --- | --- | --- | --- | --- | --- | --- | --- |
|  | **WT+S** | | | **WT+FS** | | | **TG+S** | | | **TG+FS** | | |
| Number | **1** | **2** | **3** | **1** | **2** | **3** | **1** | **2** | **3** | **1** | **2** | **3** |
| **Liver** |  |  |  |  |  |  |  |  |  |  |  |  |
| Mononuclear cell infiltration,  perivascular | 1 | 1 | 1 | 1 | 1 | 1 | 1 | 1 | 0 | 1 | 1 | 1 |
| Inflammatory foci | 1 | 1 | 0 | 1 | 1 | 1 | 1 | 2 | 1 | 1 | 1 | 0 |
| Bile duct hyperplasia | 0 | 1 | 1 | 1 | 1 | 1 | 1 | 1 | 0 | 1 | 1 | 1 |
| Oval cell hyperplasia | 1 | 1 | 1 | 1 | 1 | 1 | 0 | 1 | 0 | 1 | 1 | 1 |
| Glycogen accumulation | 0 | 0 | 1 | 0 | 1 | 0 | 3 | 3 | 3 | 2 | 2 | 2 |
| **Kidney** |  |  |  |  |  |  |  |  |  |  |  |  |
| Mononuclear cell infiltration, pelvis | 1 | 0 | 2 | 1 | 1 | 2 | 1 | 1 | 1 | 2 | 1 | 1 |
| Mononuclear cell infiltration, interstitium | 1 | 0 | 1 | 0 | 0 | 1 | 1 | 1 | 1 | 0 | 0 | 0 |
| Hyaline cast formation, tubule | 0 | 1 | 2 | 1 | 0 | 1 | 1 | 1 | 1 | 2 | 1 | 0 |
| Degeneration, with mononuclear cell infiltration, tubule, focal | 0 | 0 | 1 | 0 | 0 | 0 | 0 | 0 | 0 | 0 | 0 | 0 |
| Degeneration, tubule | 0 | 0 | 0 | 0 | 1 | 0 | 0 | 0 | 0 | 0 | 0 | 0 |
| Regeneration, tubule | 0 | 1 | 0 | 0 | 0 | 0 | 0 | 0 | 0 | 0 | 0 | 0 |

Lesions are graded from zero to five according to severity: 0=absent; 1=minimal (<1%); 2=mild (1-25%); 3=moderate (26-50%); 4=moderate severe (51-75%); 5 = severe/high (76-100%).


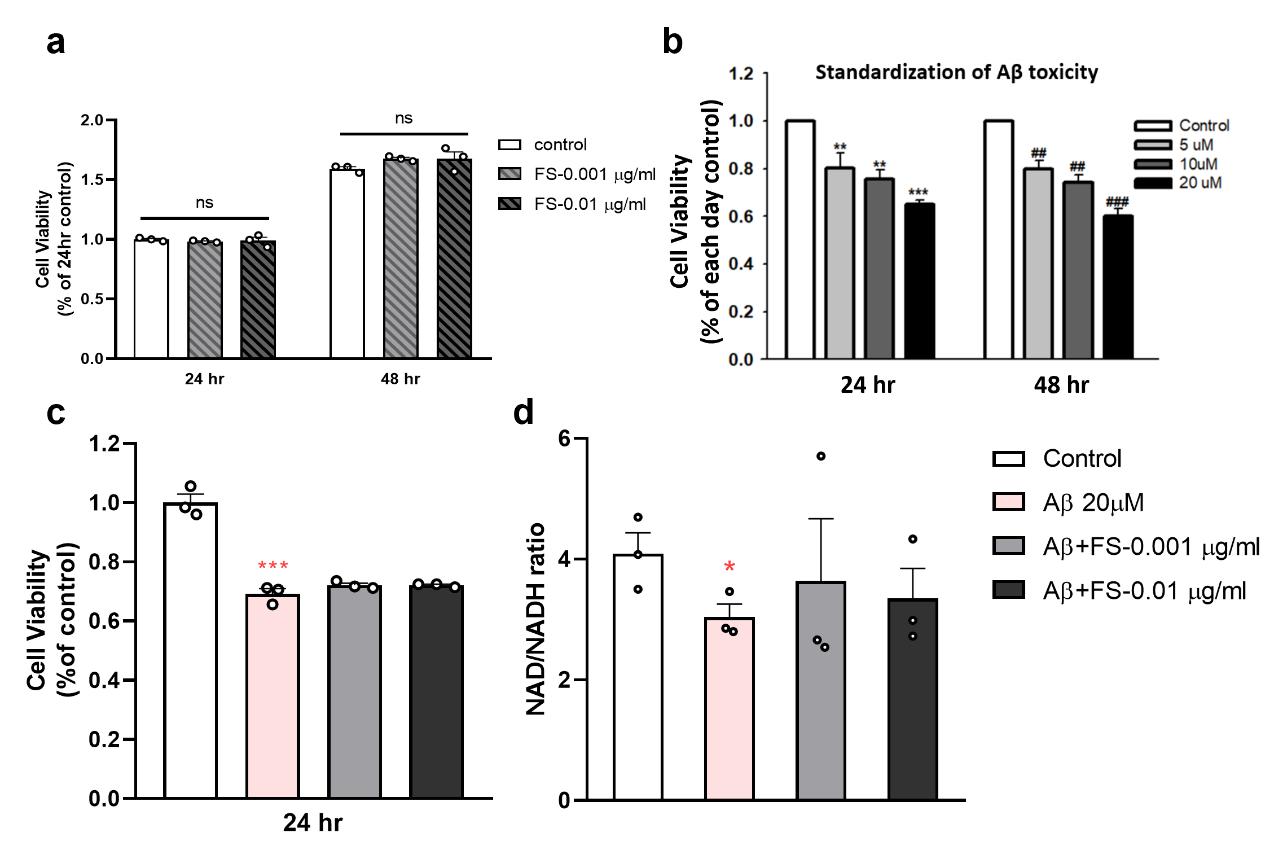


**Supplemental Figure 1.** Effects of FS on cell viability and mitochondrial activity of HT-22 cell under different treatments. (a) FS toxicity on cell viability determined by MTT assay. (b) Neurotoxicity of HT 22 cells under different Aβ concentrations determined by MTT assay. (c) Cell viability under Aβ with or without treatment with FS. (d) NAD/NADH ratio of cells under Aβ with or without treatment with FS. **p* < 0.05, ***p* < 0.01, ****p* < 0.001 compared to 24-hr control group; ##*p* < 0.01; ###*p* < 0.001 compared to 48-hr control group (n=3).


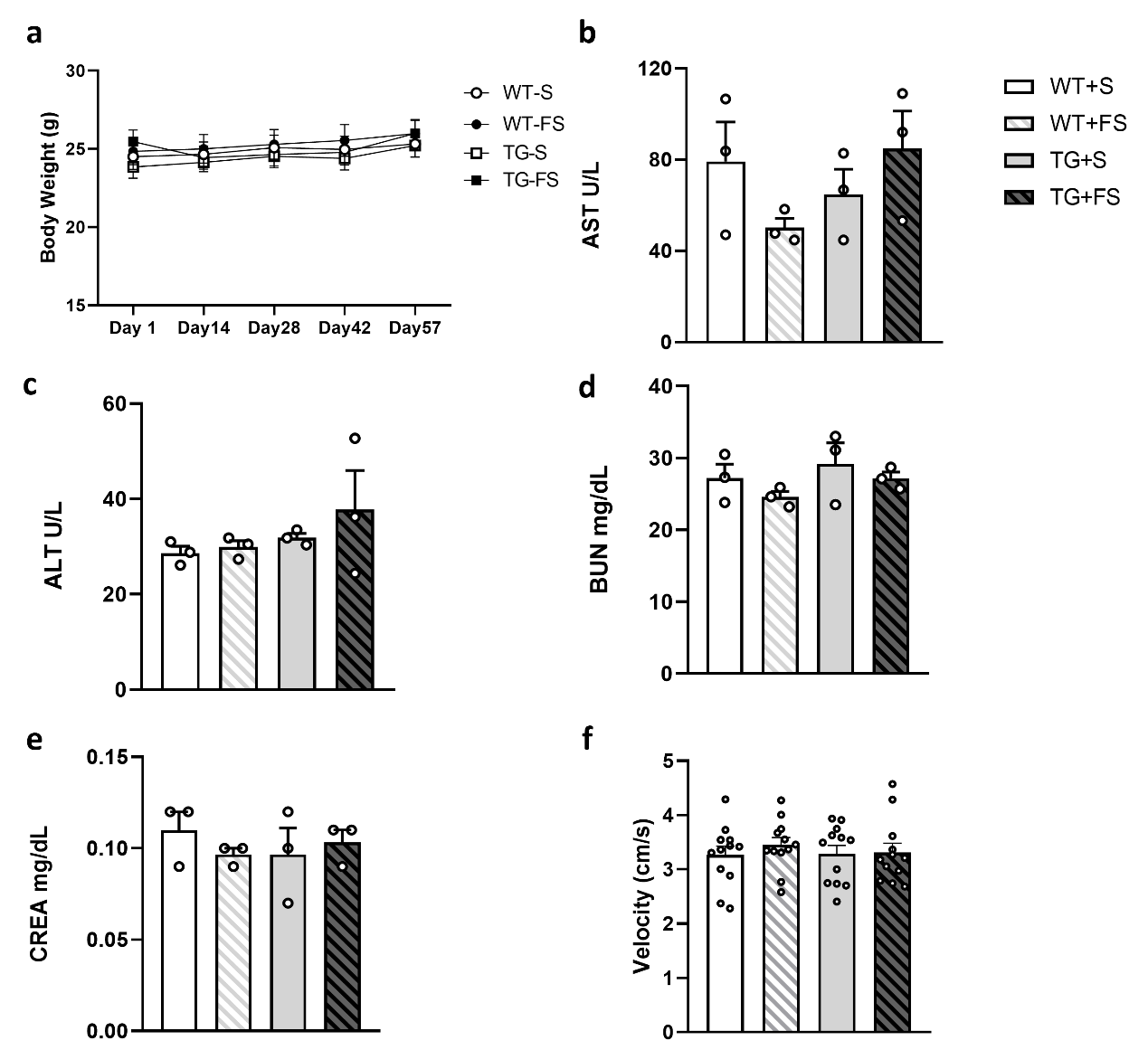


**Supplemental Figure 2.** Evaluation of the toxicity of FS in mice. (a) The body weight of mice was measured during the experimental process. Mouse (n = 3) serum biochemical levels of aspartate aminotransferase (AST/GOT) (b) and alanine aminotransferase (ALT/GPT) (c), blood urea nitrogen (BUN) levels (d), and serum creatinine (CREA) (e). (f) The mouse velocity was analyzed in OFT (n = 12).


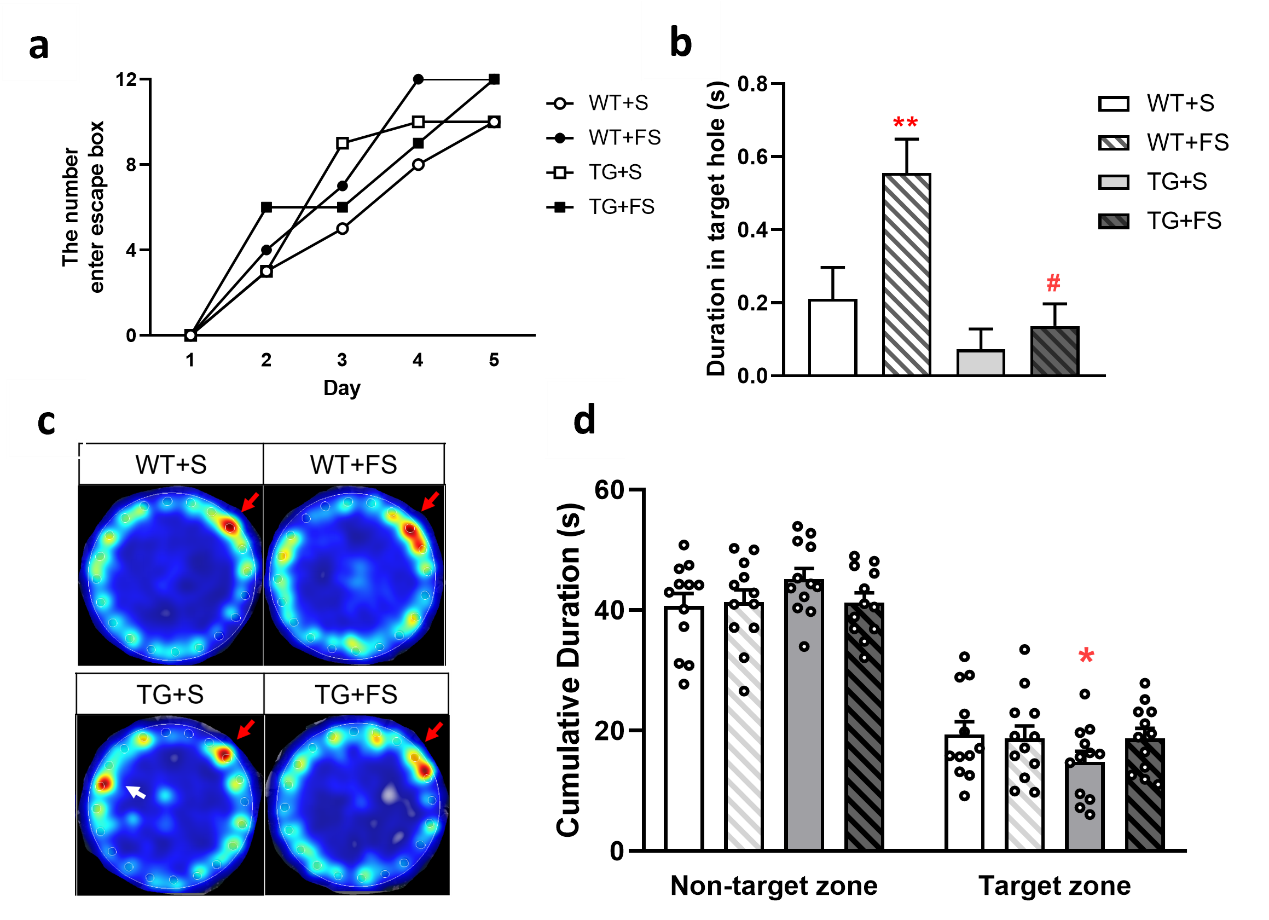


**Supplemental Figure 3.** Effect of FS on BM. (a) Number of mice successfully entering the escape box during BM training. (b) The duration of mice stayed in the target hole within the first min of the BM probe 1. (c) Heatmap showing mice location during the BM probe 1. Red arrows indicate the target hole, while white arrow points to the intense incorrect location in TG+S group. (d) Time spent in the target and non-target areas during the first min of the BM probe 1. **p* < 0.05, ***p* < 0.01 compared to WT+S group; #*p* < 0.05 compared to TG+S group (n = 12).

**
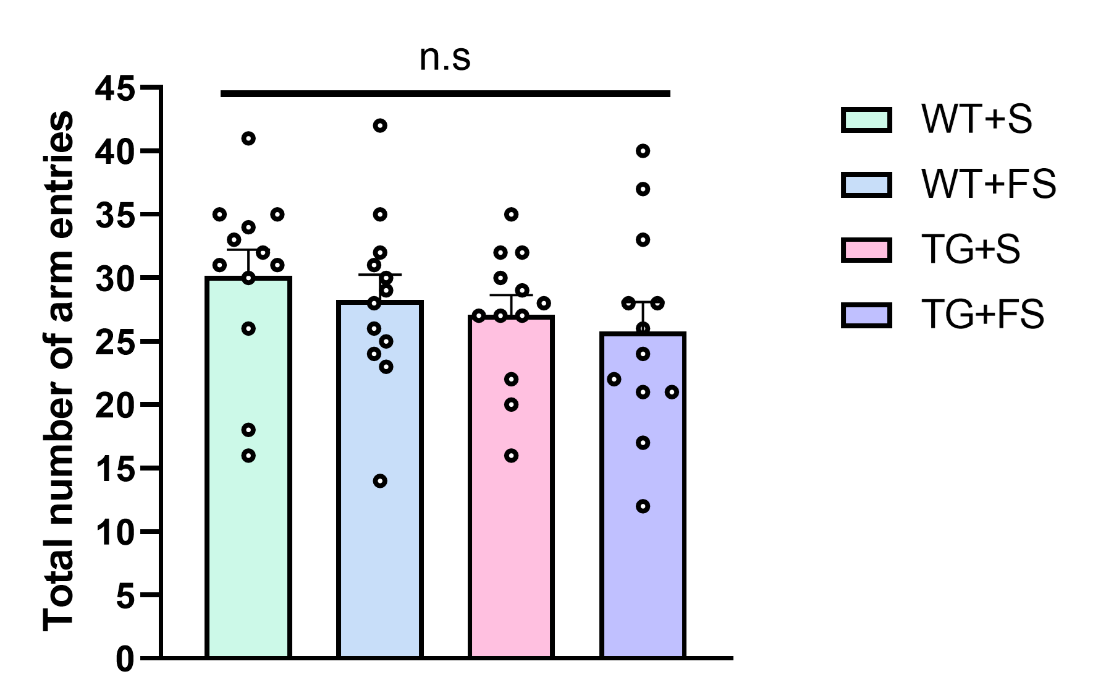
**

**Supplemental Figure 4.** The number of arm entries in the Y-maze between the 4 groups (n = 12).


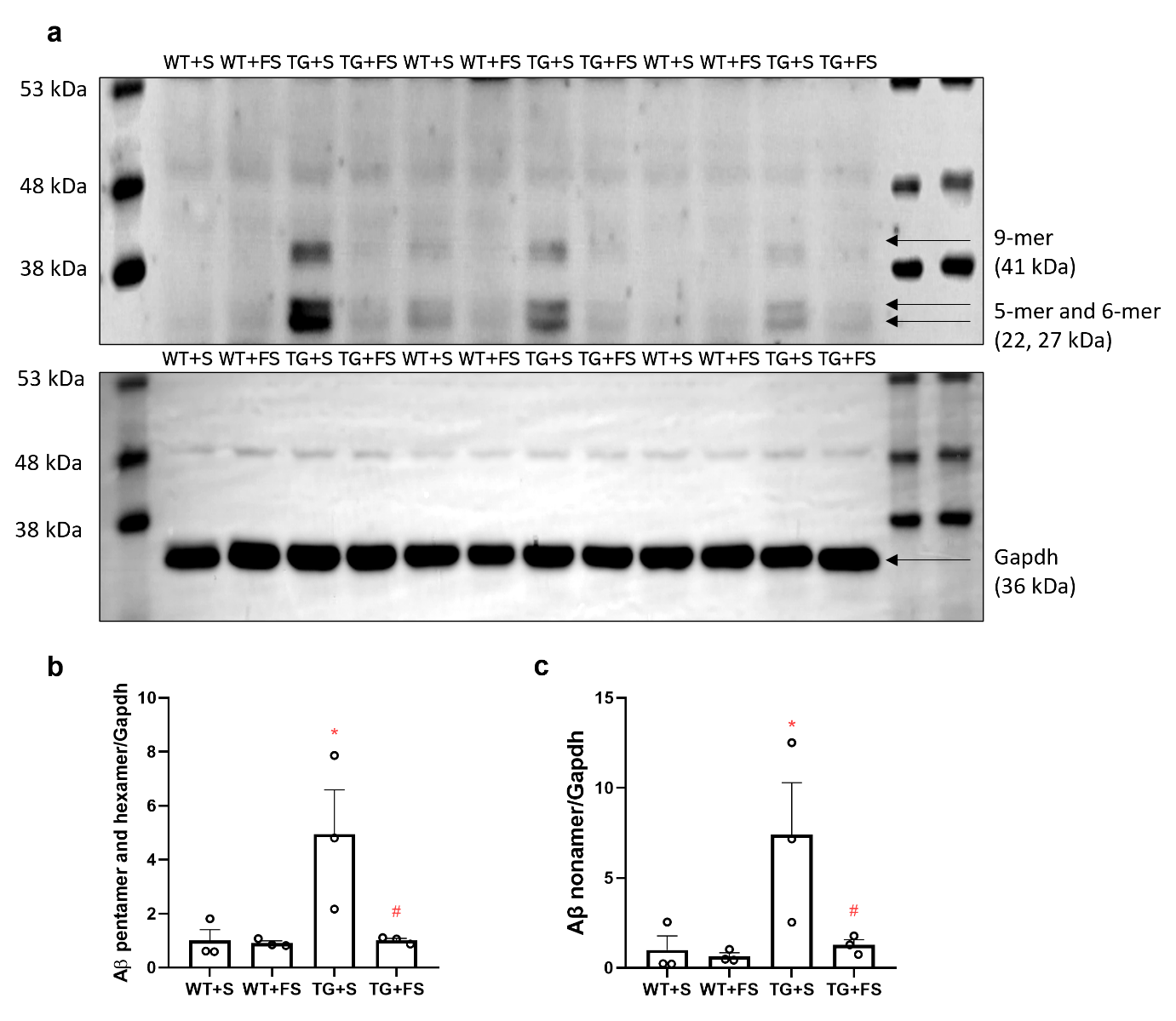


**Supplemental Figure 5.** FS reduces the Aβ oligomers in 5xFAD hippocampus. (a) Results of WB analyses of anti-beta Amyloid (H31L21). The statistical analysis of Aβ pentamer and hexamer (b) and Aβ nonamer (c). **p* < 0.05, ***p* < 0.01 compared to WT+S group; #*p* < 0.05 compared to TG+S group (n = 3).


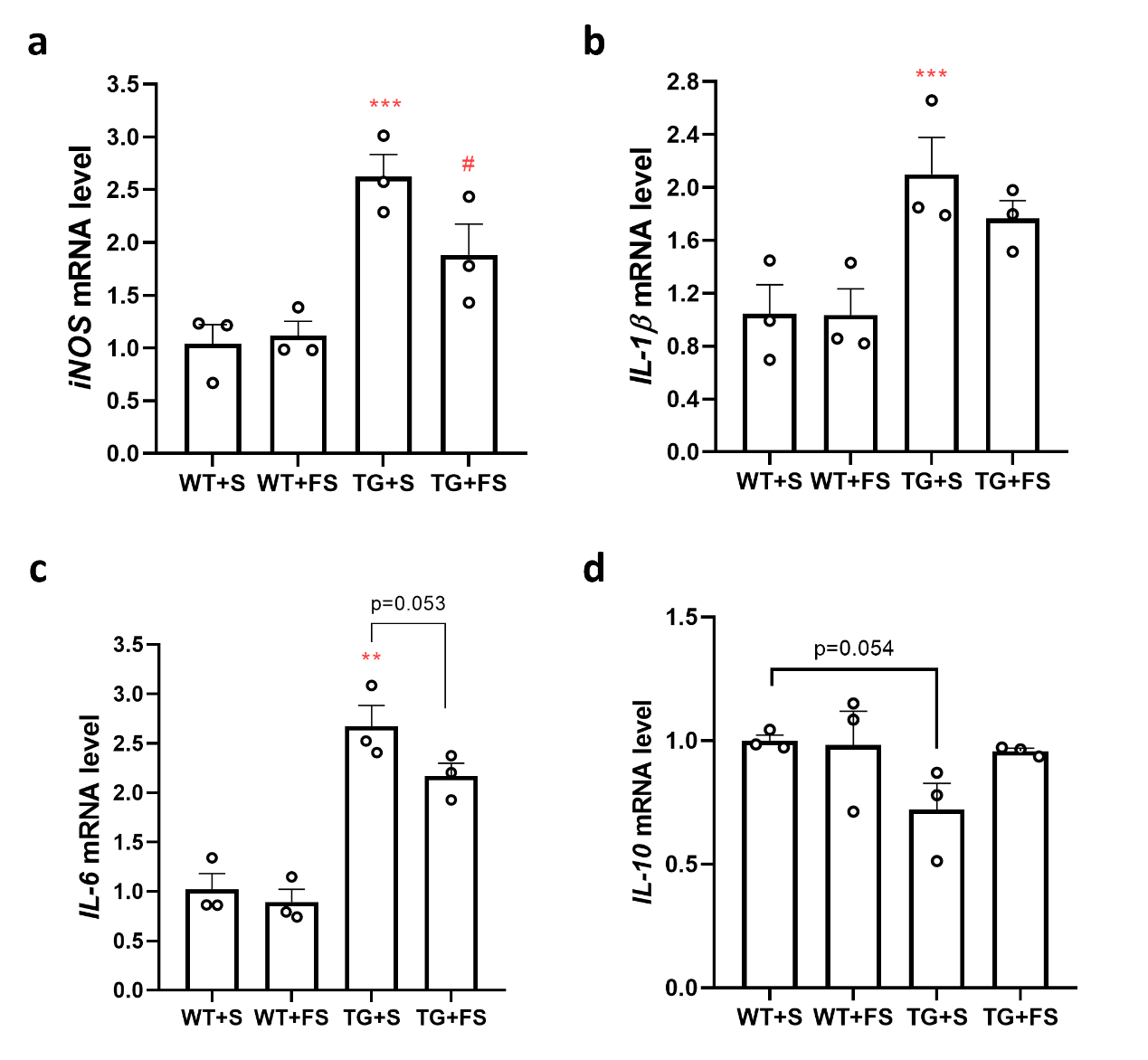


**Supplemental Figure 6.** The mRNA expression in mouse hippocampus, including: (a) *iNOS*, (b) *IL-1β*, (c) *IL-6*, and (d) *IL-10*. ***p* < 0.01, ****p* < 0.001 compared to WT+S group; #*p* < 0.05, compared to TG+S group (n = 3).


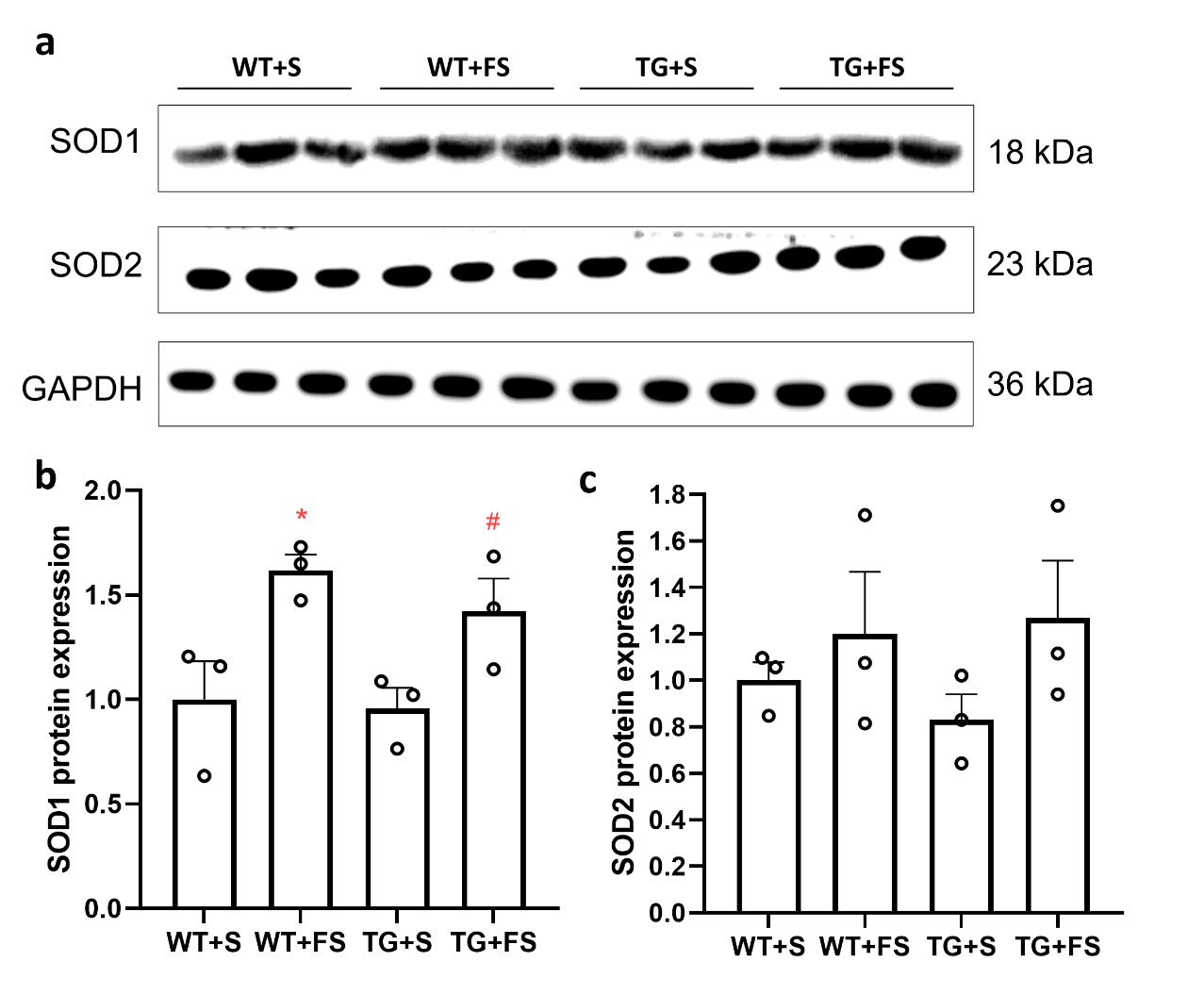


**Supplemental Figure 7.** (a) Expression of SOD1 and SOD2 in the mouse hippocampus analyzed by WB. (b) The relative level of SOD1 was normalized to Gapdh. (c) The relative level of SOD2 was normalized to Gapdh **p* < 0.05 compared to WT+S group; *#p* < 0.05 compared to TG+S group (n = 3).
