# Supplementary material for: Fermented Soybean Pulp Alleviates Disease Progression of 5×FAD Model Mice
Source: Mol Neurobiol. 2025 Jul 15;62(11):14121–39. doi: 10.1007/s12035-025-05191-y (PMC12511221; doi:10.1007/s12035-025-05191-y)
Supplement: Supplementary file 2 — Supplementary Material 2 (DOCX 23.2 MB) [file 12035_2025_5191_MOESM2_ESM.docx]

**Supplementary Material**

We acknowledge that the membrane was cropped due to budget constraints, which was necessary to minimize protein extraction and reduce antibody consumption. This approach allowed us to use smaller chambers and conserve antibodies. For research transparency, we have provided the raw data (N=3) below.

Raw Data For Figure 3 (N=3)




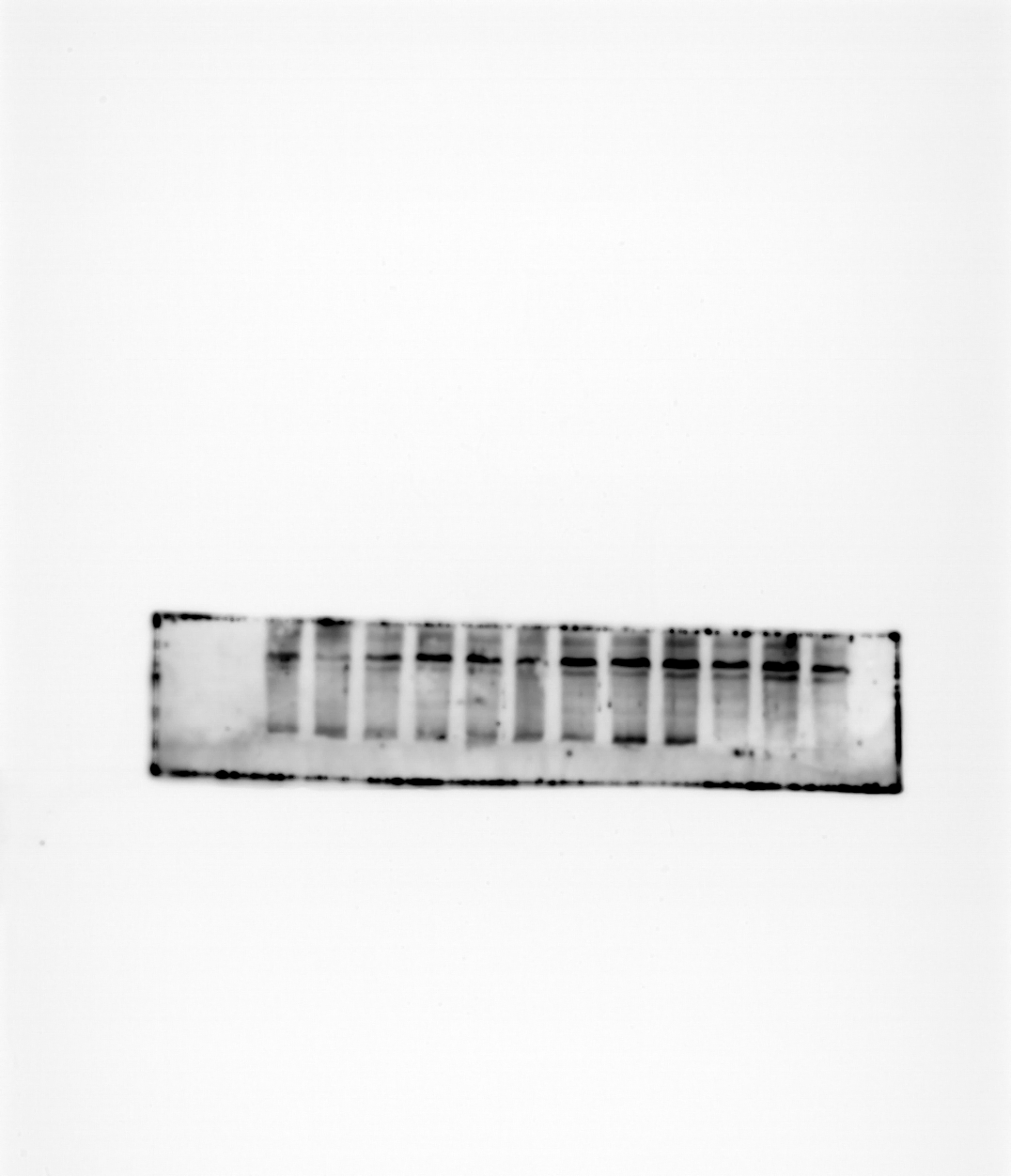

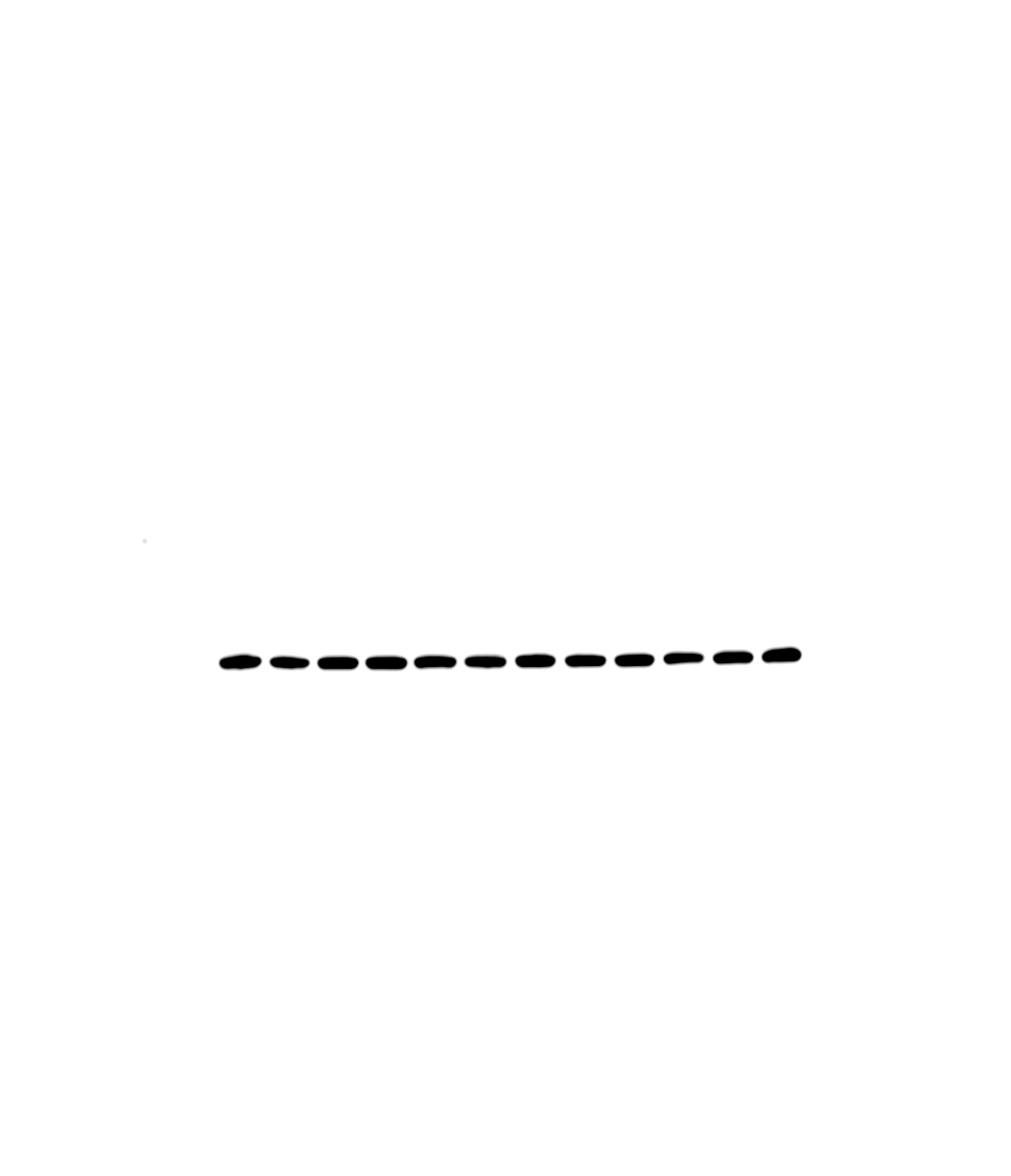


WT+S

WT+FS

TG+FS

TG+S

WT+S

WT+FS

TG+FS

TG+S

WT+S

WT+FS

TG+FS

TG+S

Gapdh

(36 kDa)

Bace1

(56 kDa)

IDE

(130 kDa)

Raw Data For Figure 4 (N=3)


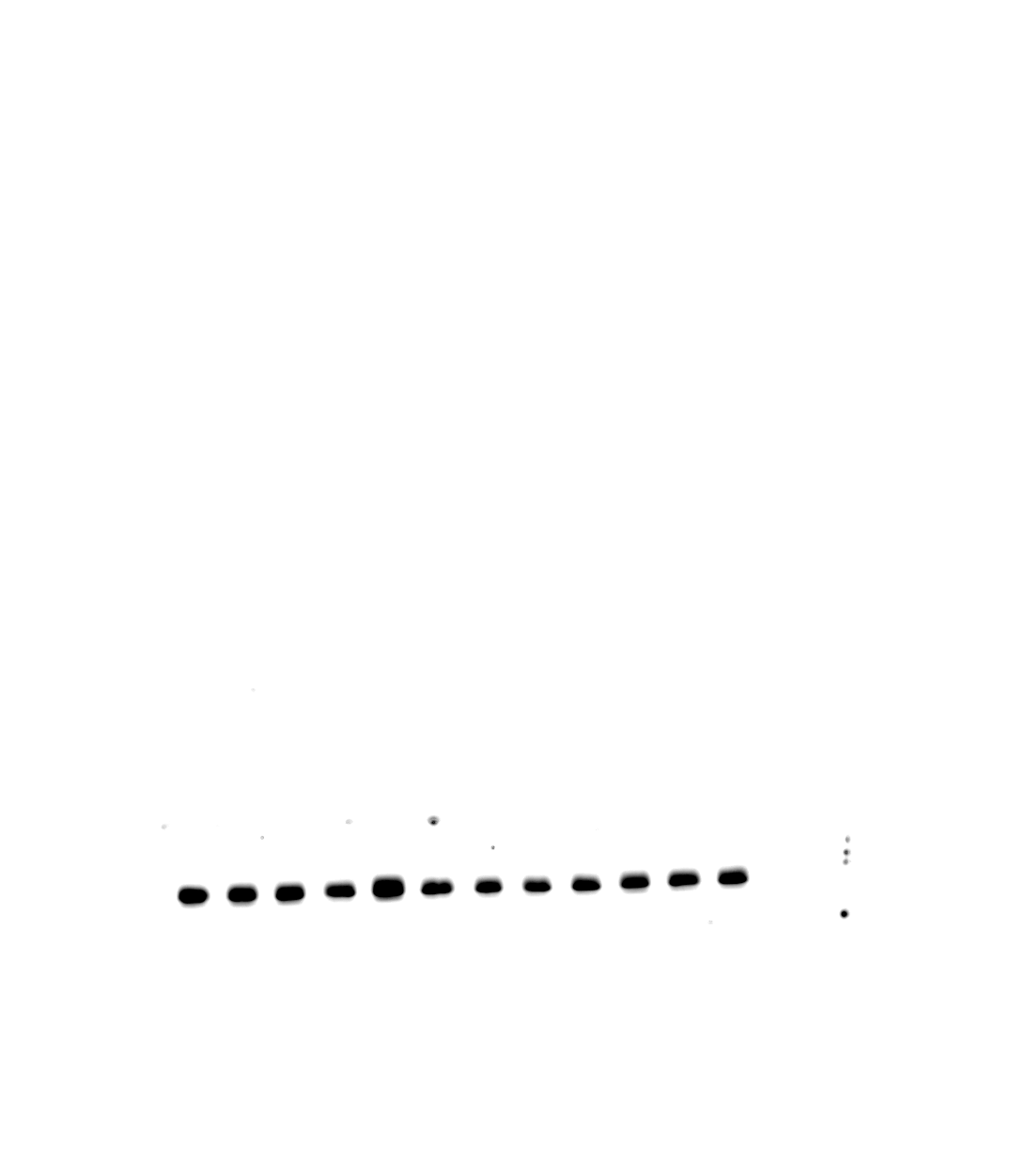

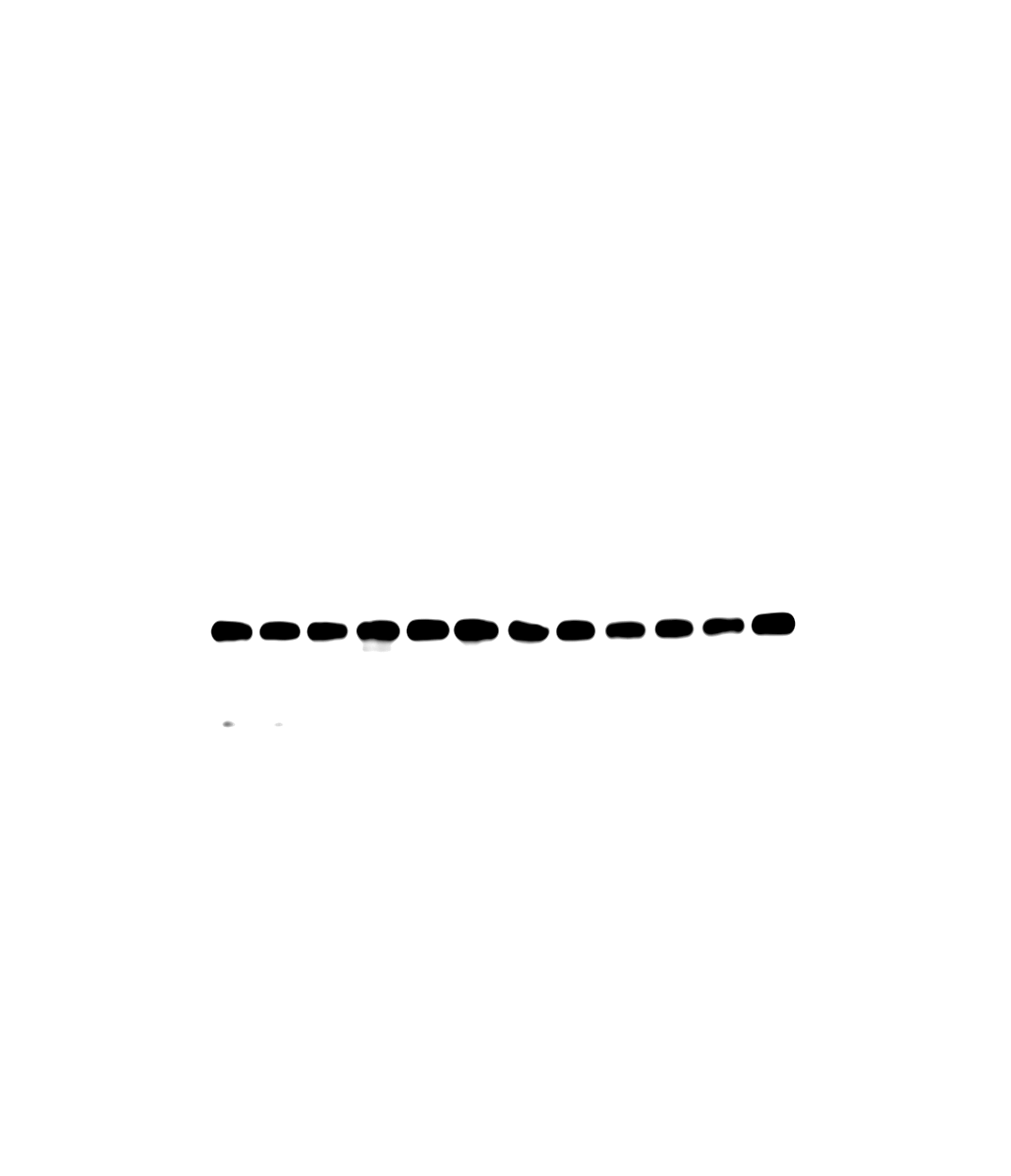

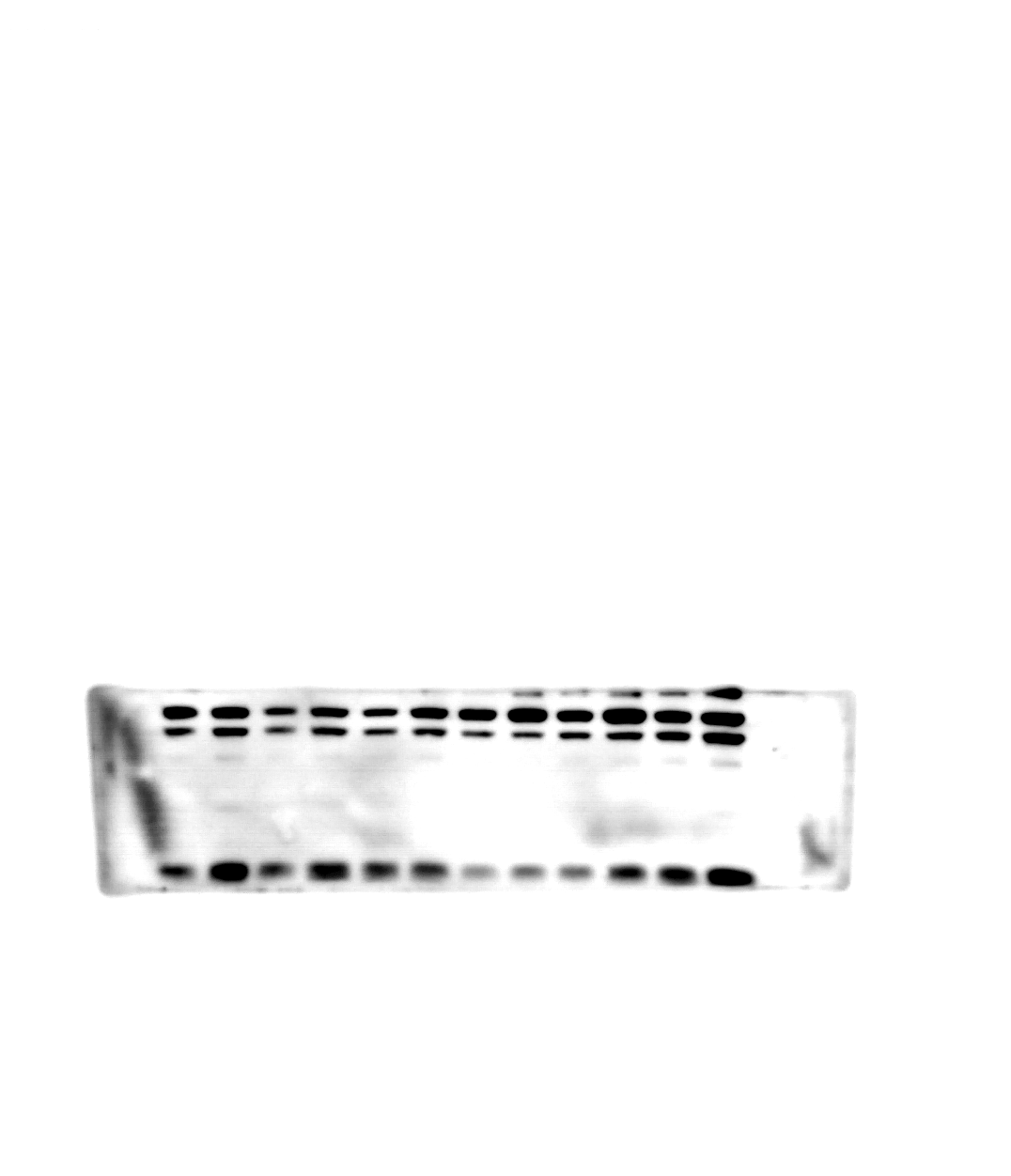




mature BDNF

(13 kDa)

pro BDNF

(32 & 28 kDa)

Gapdh

(36 kDa)

Synaptophysin

(34 kDa)

PSD95

(95 kDa)

WT+S

WT+FS

TG+FS

TG+S

WT+S

WT+FS

TG+FS

TG+S

WT+S

WT+FS

TG+FS

TG+S

WT+S

WT+FS

TG+FS

TG+S

Raw Data For Figure 5 (N=3)


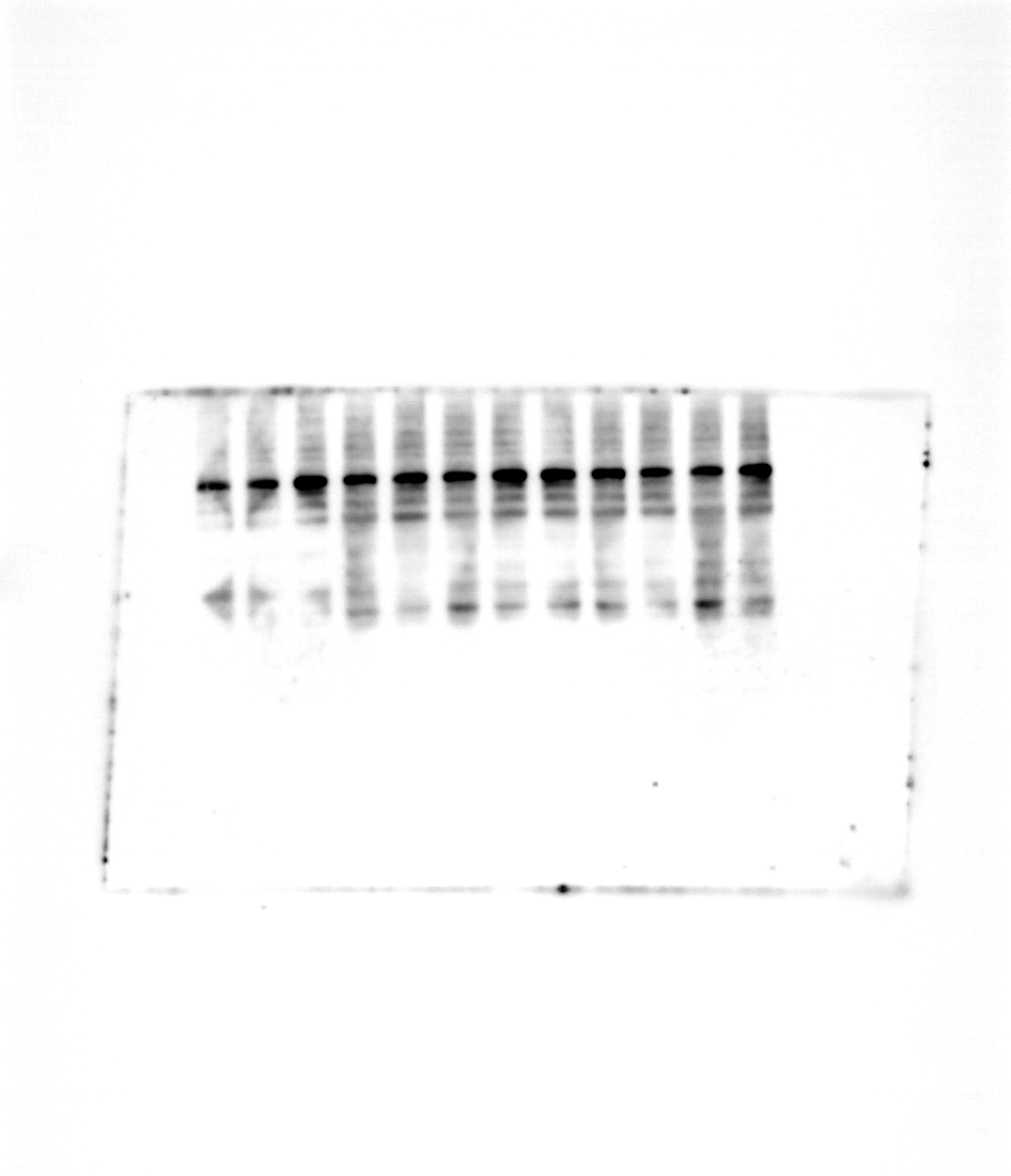

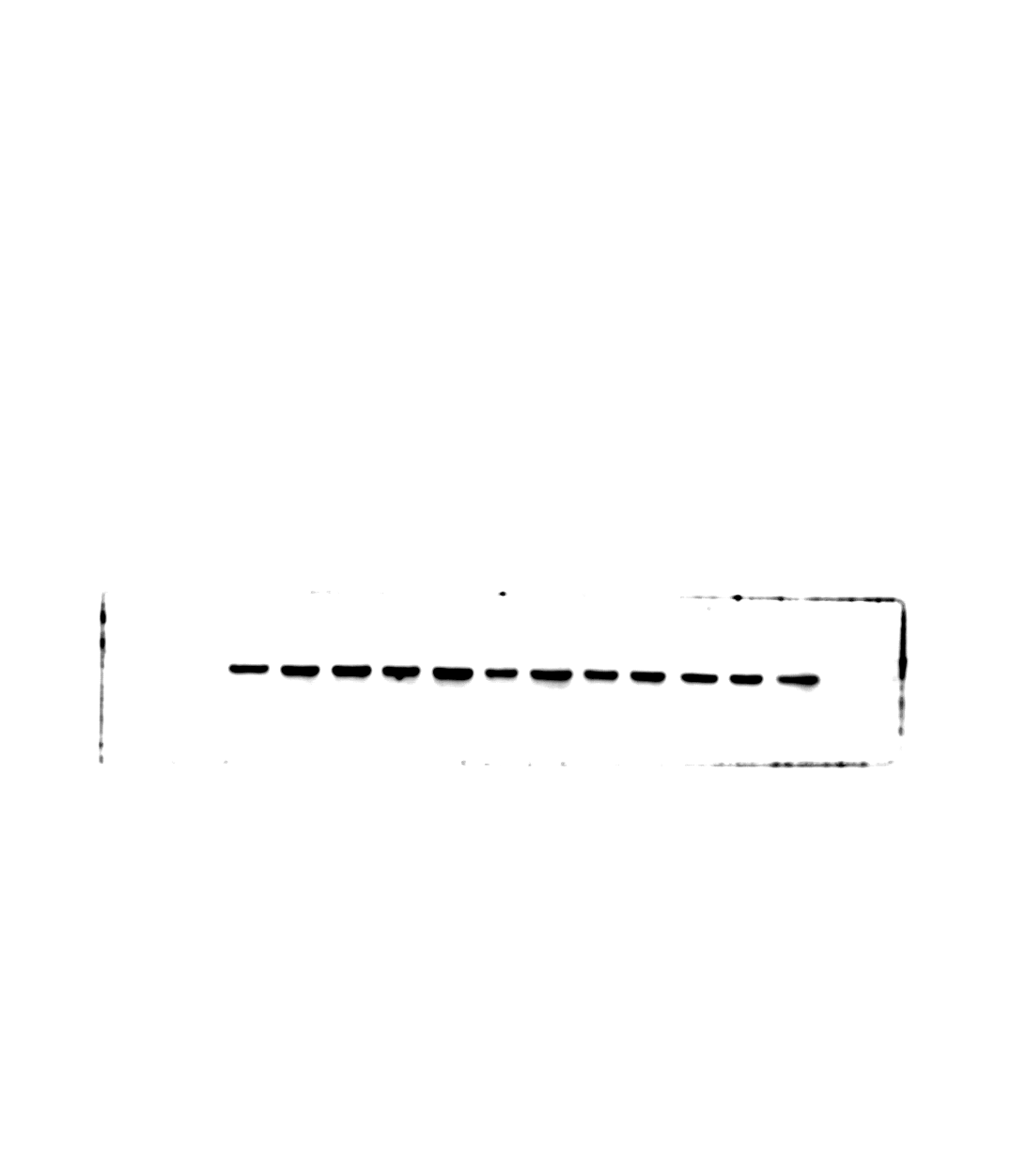


WT+S

WT+FS

TG+FS

TG+S

WT+S

WT+FS

TG+FS

TG+S

p65

(65 kDa)

p-p65

(65 kDa)

Raw Data For Figure 6 (N=3)


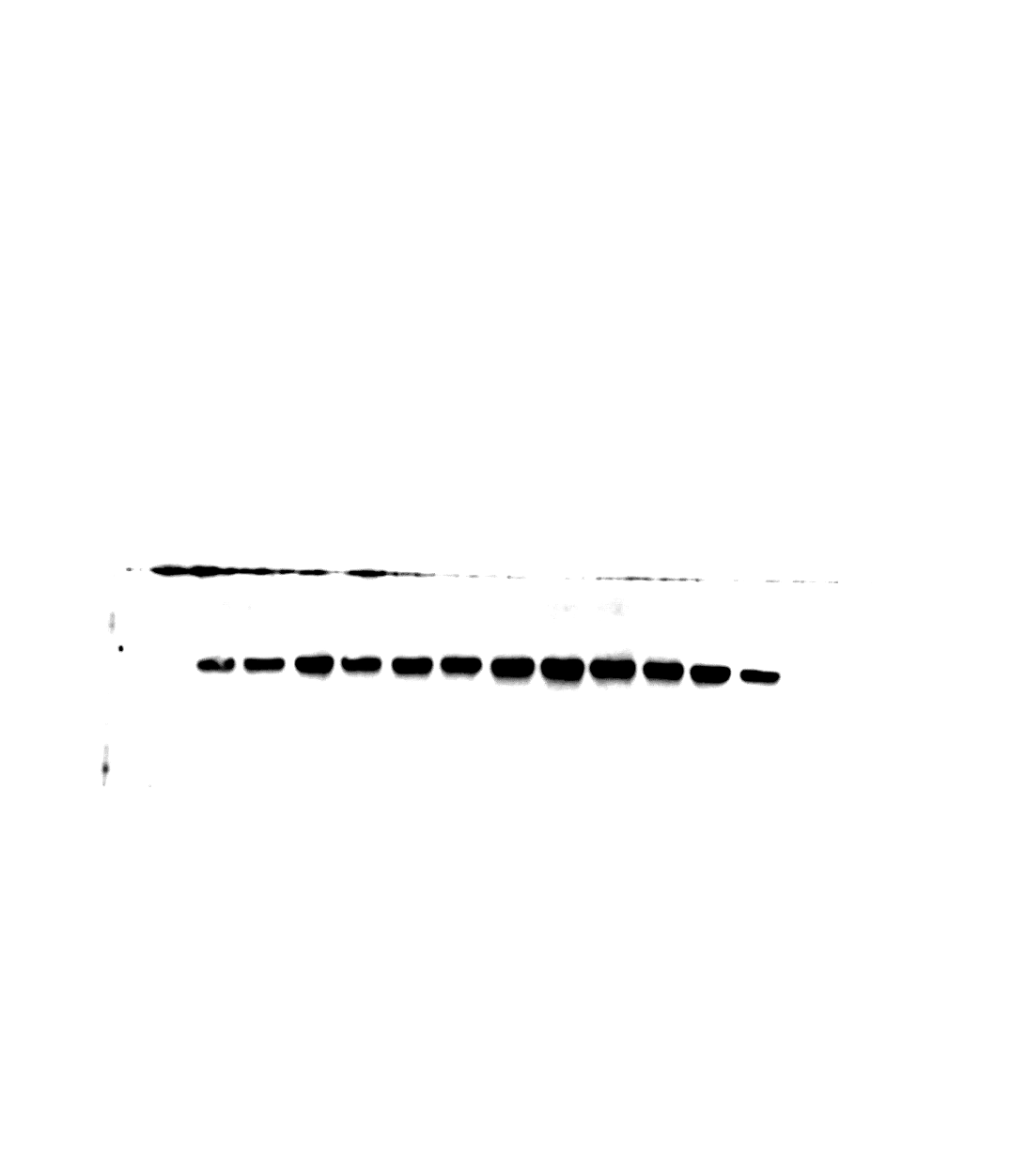





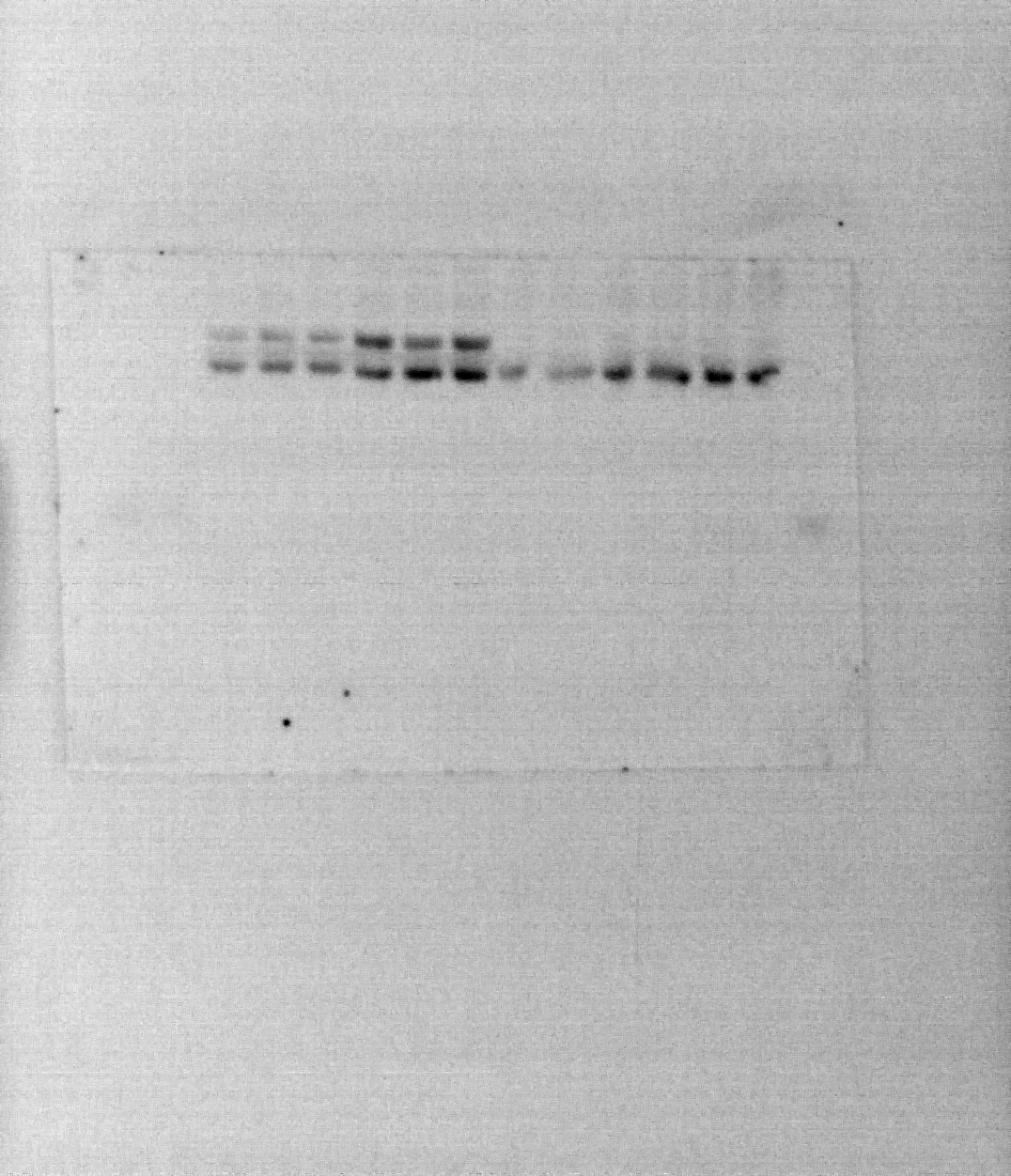


Cleaved-CASP3

(17 kDa)

CASP3

(35 kDa)

WT+S

WT+FS

TG+FS

TG+S

WT+S

WT+FS

TG+FS

TG+S

Gapdh

(36 kDa)

Bcl-2

(26 kDa)

WT+S

WT+FS

TG+FS

TG+S

WT+S

WT+FS

TG+FS

TG+S

Bax

(21 kDa)


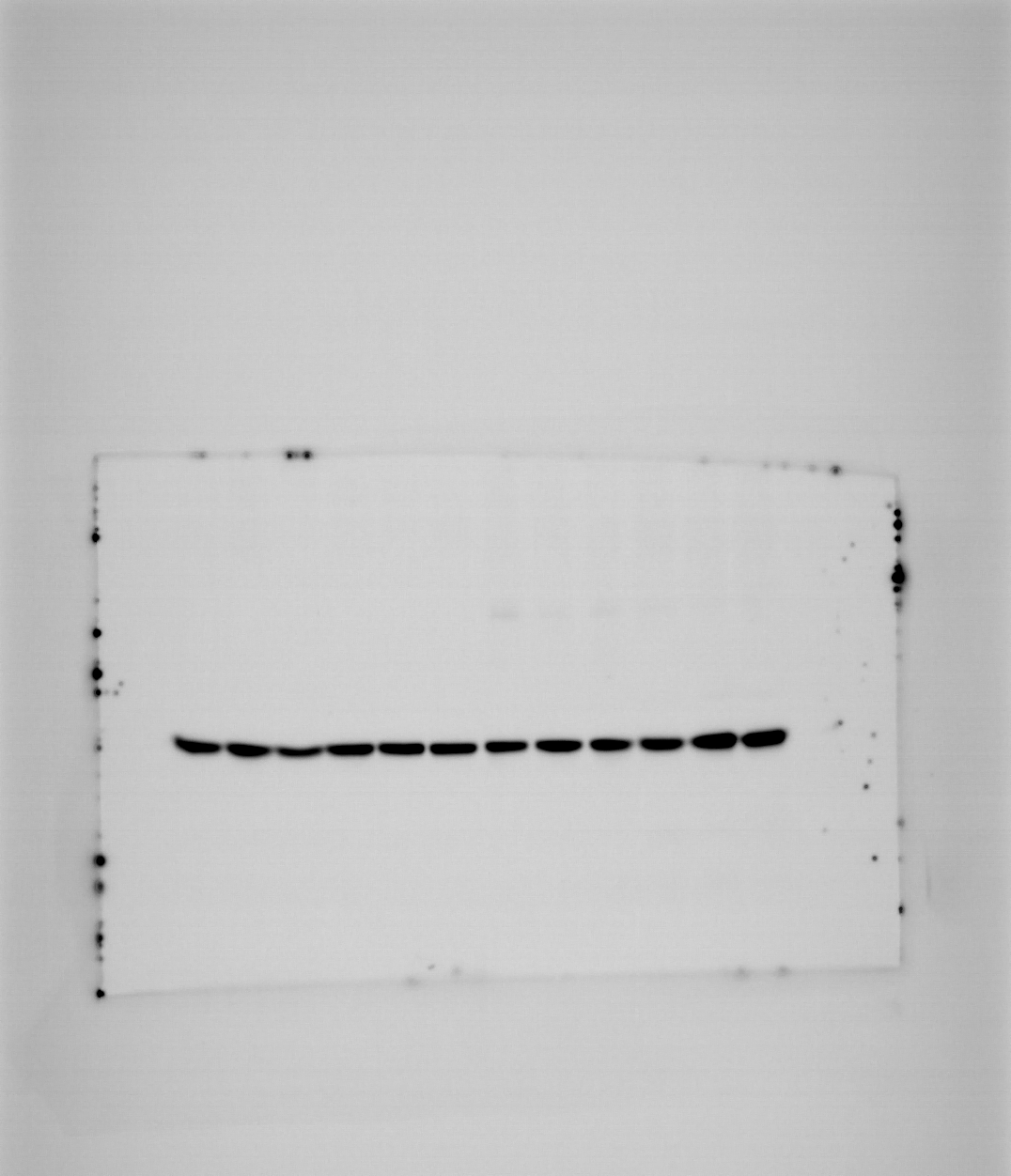


WT+S

WT+FS

TG+FS

TG+S

Gapdh

(42 kDa)

Raw Data For Figure 7 (N=3)


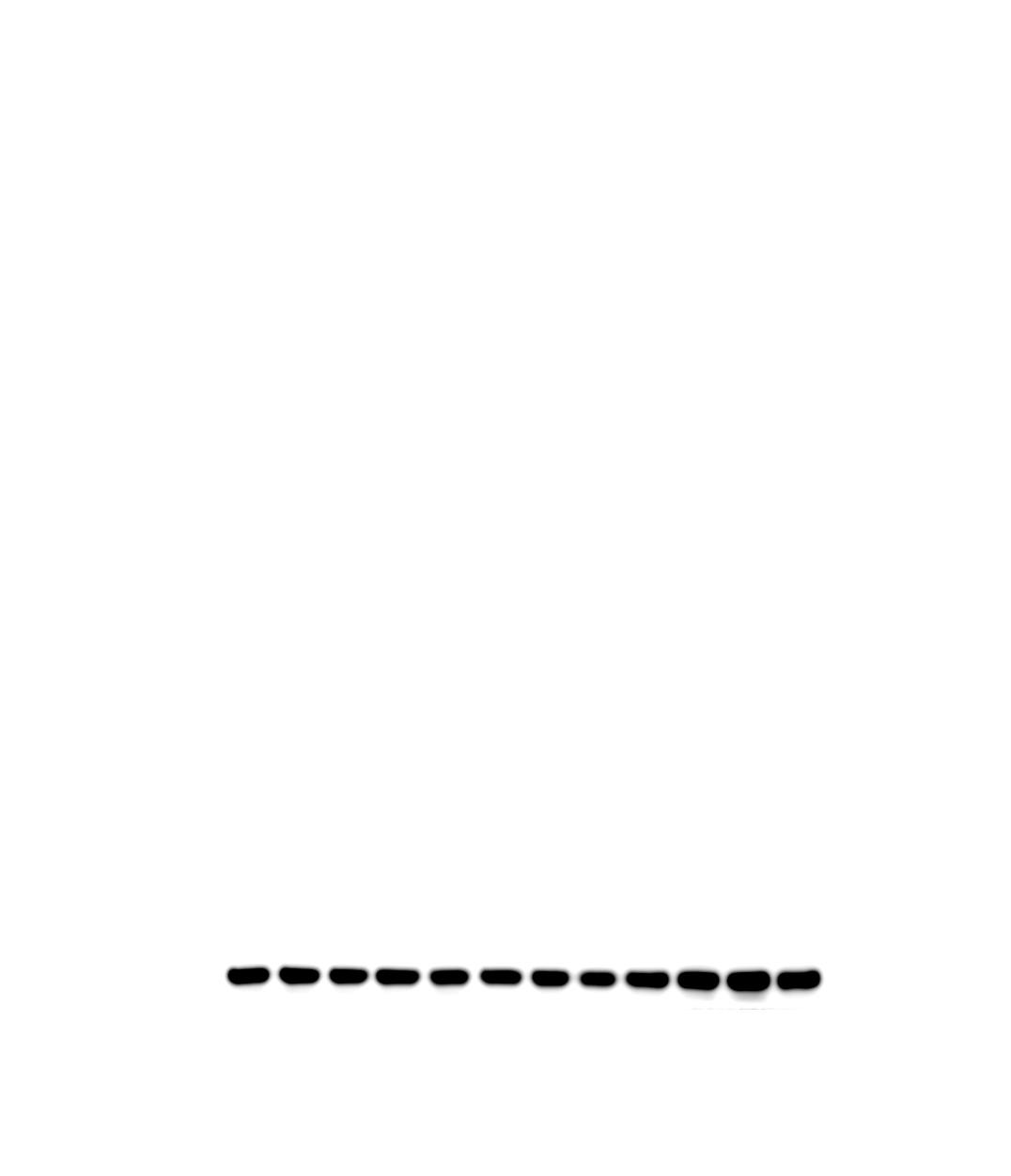

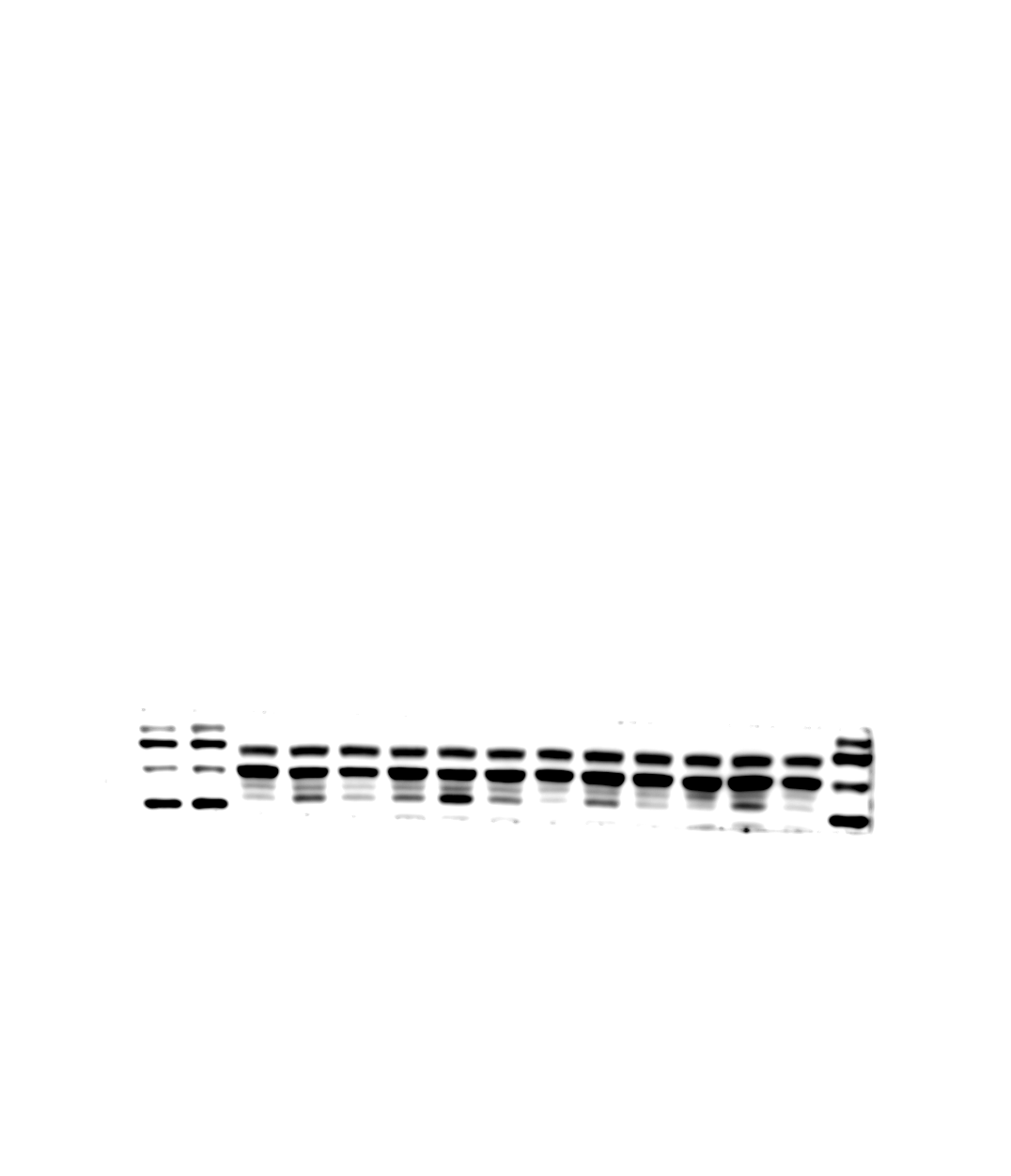










93 kDa

Akt

(56 kDa)

WT+S

WT+FS

TG+FS

TG+S

WT+S

WT+FS

TG+FS

TG+S

WT+S

WT+FS

TG+FS

TG+S

M M

M

WT+S

WT+FS

TG+FS

TG+S

p-GSK3β (Ser9)

(46 kDa)

GSK3β

(46 kDa)

42 kDa

70 kDa

36 kDa

p-Akt

(56 kDa)

WT+S

WT+FS

TG+FS

TG+S

Nrf2

(100 kDa)

Gapdh

(36 kDa)

WT+S

WT+FS

TG+FS

TG+S

Raw Data For Figure S5 (N=3)

53 kDa

M WT+S WT+FS TG+S TG+FS WT+S WT+FS TG+S TG+FS WT+S WT+FS TG+S TG+FS M M


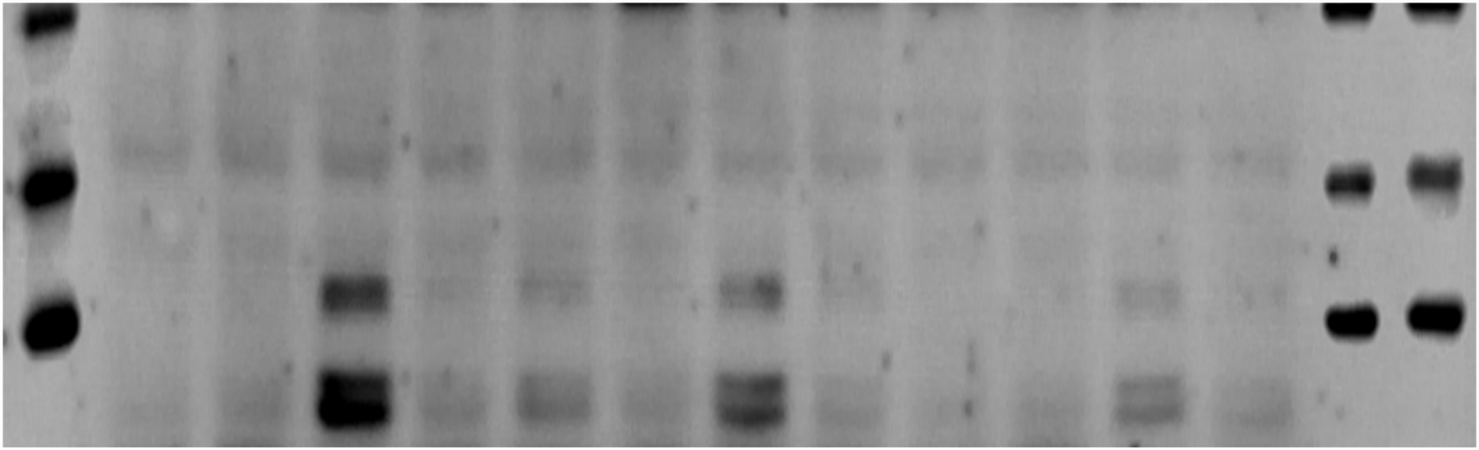


48 kDa

38 kDa

9-mer (41 kDa)

6-mer (27 kDa)

5-mer (22 kDa)

53 kDa

M WT+S WT+FS TG+S TG+FS WT+S WT+FS TG+S TG+FS WT+S WT+FS TG+S TG+FS M M


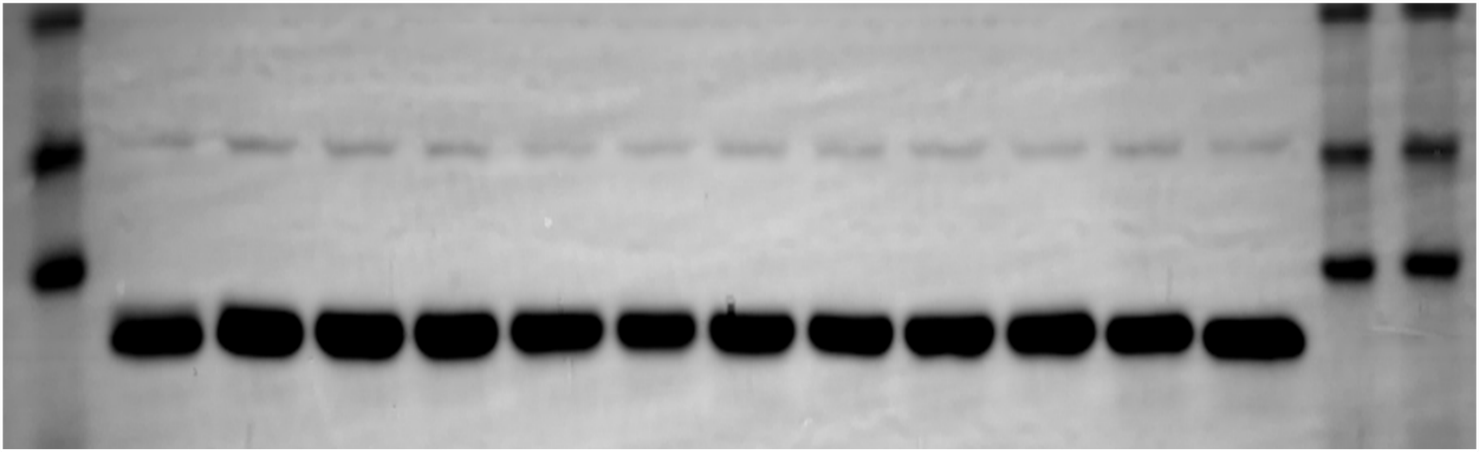


Gapdh

(36 kDa)

48 kDa

38 kDa

Raw Data For Figure S7 (N=3)




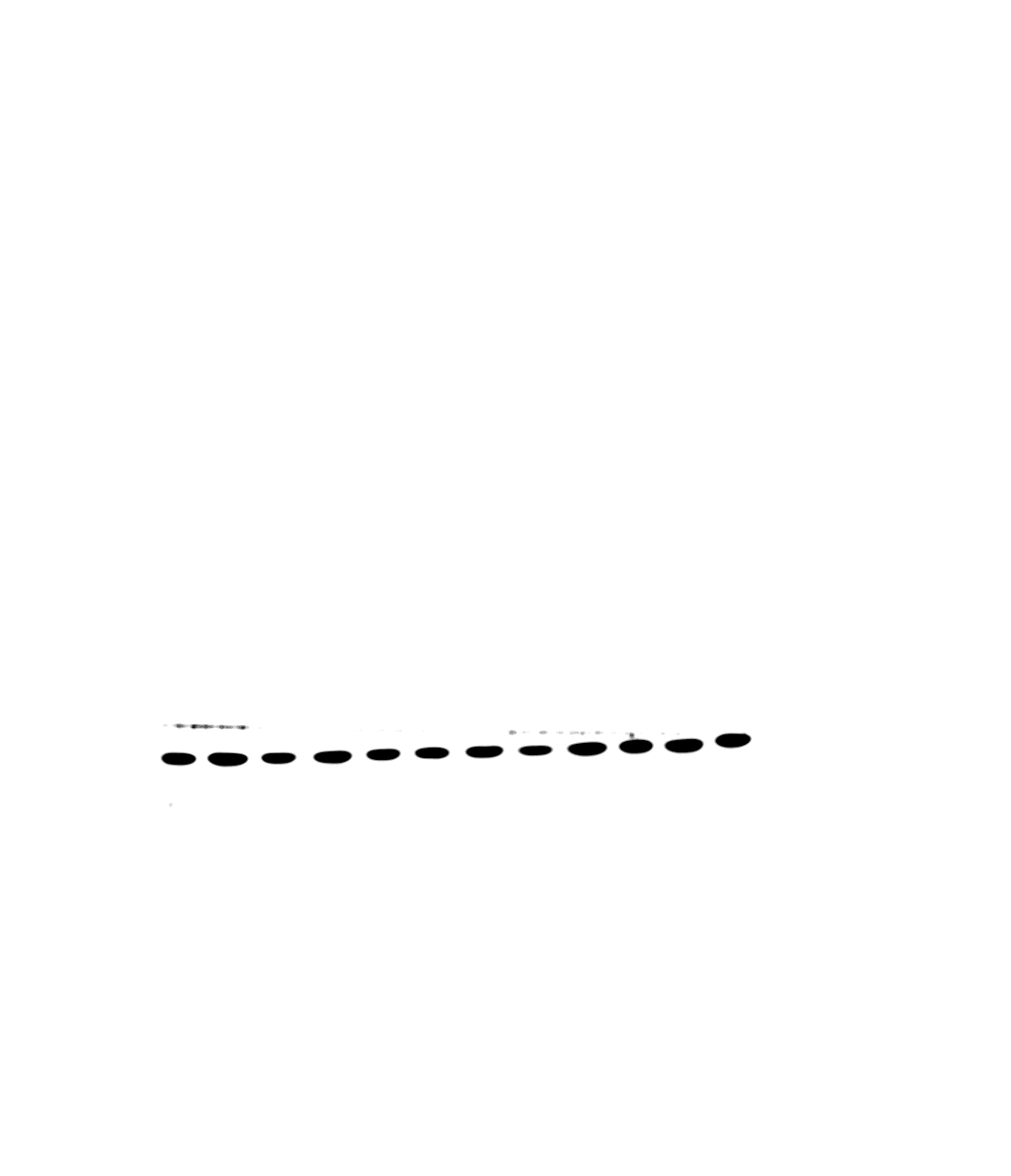

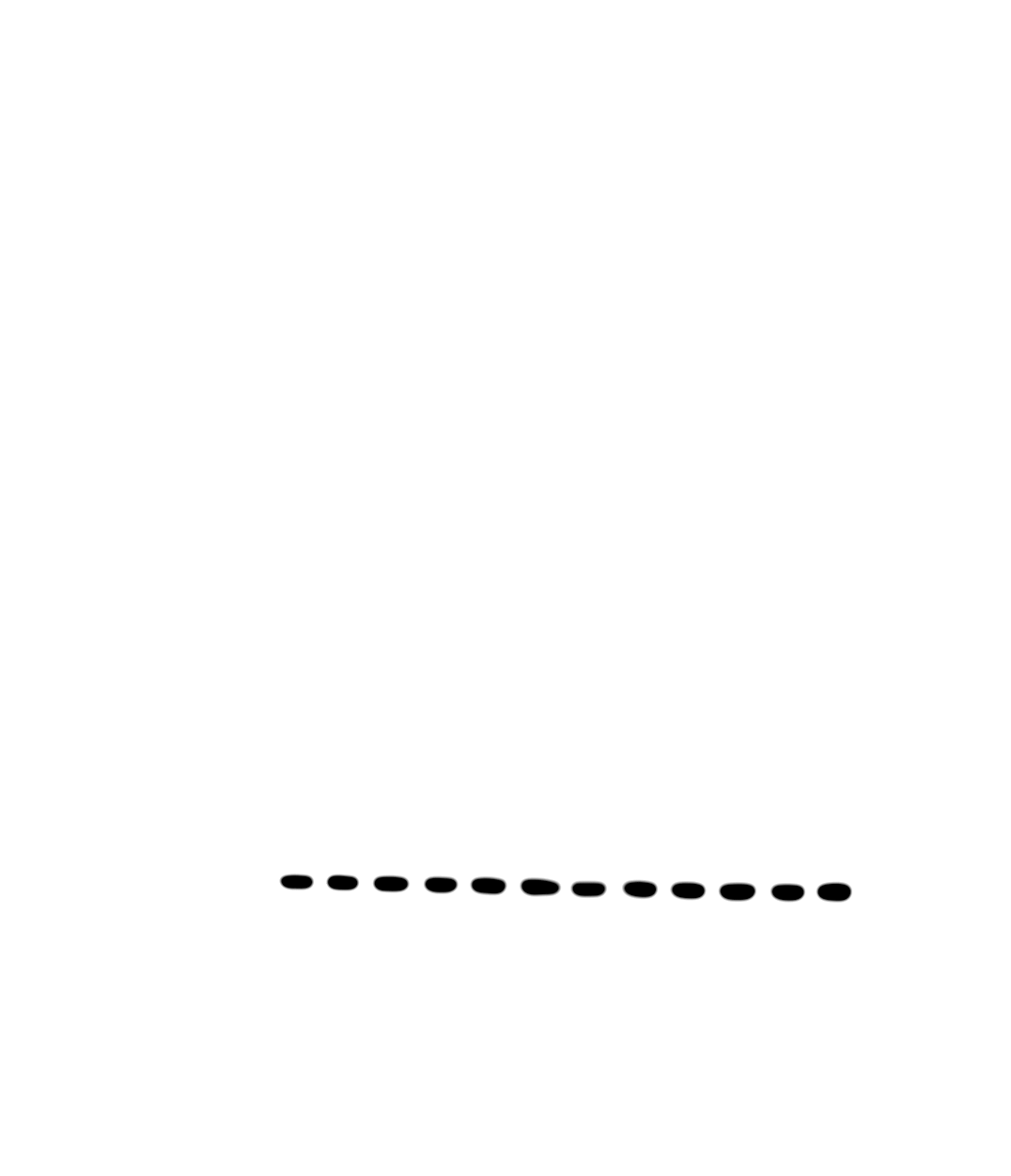


Gapdh

(36 kDa)

SOD2

(22 kDa)

SOD1

(18 kDa)

WT+S

WT+FS

TG+FS

TG+S

WT+S

WT+FS

TG+FS

TG+S

WT+S

WT+FS

TG+FS

TG+S
